# Supplementary material for: Mobilising social support to improve mental health for children and adolescents: A systematic review using principles of realist synthesis
Source: PLoS One. 2021 May 20;16(5):e0251750. doi: 10.1371/journal.pone.0251750 (PMC8136658; doi:10.1371/journal.pone.0251750)
Supplement: S1 Table — (DOCX) [file pone.0251750.s002.docx]

Table S1: Assessment of relevance for included studies

| Study ID | Assessment | Rating |
| --- | --- | --- |
| Asghar et al (2018) | Increasing social support is a programme goal but social support is not defined or conceptualised; no programme theory reported; standardised measures for child wellbeing (self-esteem, confidence) but not for social support | Low |
| Ayton and Joss (2016) | Social support is specific programme goal but not defined or conceptualised; only limited theory about how to increase social support and link between social support and child and health wellbeing | Low |
| Bohleber et al (2016) | Increasing social support is specific programme goal; social support and child outcomes measured with standardised scales; consistent between hypotheses and tested relationships on social support and child outcomes but not much detail on program theory and mechanisms | Moderate |
| Byrne et al (2012) | Social support not primary programme goal; strong social support conceptualisation; comprehensive social support measure; child outcomes only measured through perceptions of child development and child rearing (no standardised measure) | Moderate |
| Branch et al (2013) | Increasing social support is primary programme goal; social support not well conceptualised and only refers to formal support provided by schools; mechanisms and theories do not directly refer to social support; child outcomes primarily refer to school related outcomes | Low |
| Cho et al (2013) | Study hypothesises important role of social support (and measures this); however, social support not part of programme theory; whilst social support measure included informal and formal support, the only social support source targeted by the intervention is support from healthcare professionals | Low |
| Cluver et al (2017) | Theory of change (depicted) includes social support but no further conceptualisation of social support; no clear programme theory; no information about expected mechanisms by which intervention would increase social support and in turn improve child outcomes; social support and child outcomes measured with standardised scales (only child behaviour and no other child mental health outcomes measured) | Low |
| Deutsch et al (2017) | Social support (conceptualised in form of mentor and peer support and relationships with friends and families) is primary goal of the programme; mechanism of how the intervention might increase social support and their refinement is subject to this study; not much detail about how social support is expected to improve child outcomes | Moderate |
| DeWit et al (2016) | Increasing social support is a programme goal but social support not defined or conceptualised; programme theory aims match well with hypothesised mechanisms, which are based on theory and evidence and well described; and also addressed in findings and conclusions; standard measures for social support and child outcomes | Moderate |
| Doty et al (2017) | Strong in conceptualisation of social support, including hypothesised pathways to social support and pathways for relationship between social support and child outcomes; however, fairly abstract with no clear explanation how this relates to a program logic or theory | Moderate |
| Drummond et al (2014) | Social support mainly refers to linkages to formal system, which is the main outcomes measure; link to informal support is made (e.g. linkages to services and school expected to increase social support for family and child) but not explained with much detail | Moderate |
| Eddy et al (2017) | Whilst authors explain social support as important intervention goal and a key mechanism for improved child mental health and development outcomes, it is not measured through standardised perceived social support scale; authors refer to this in the discussion section as possible limitation; child outcome measure via standardised mental health scales | Moderate |
| Hauken et al (2015) | Increasing social support is the primary goal of the study; social support is well conceptualised and measured using standardised scale; clear hypotheses in relation to social support and relationship with child outcomes, which are based on theory and evidence; child outcome measured with standardised scales | High |
| Ingram et al (2015) | Social support is a primary programme goal and conceptualised well (although without a definition); mechanisms provided as to how social support is changed through the intervention and how it is expected to lead to improved child outcomes | Moderate |
| January et al (2016) | Social support is a specific program aim; choice of social support measure closely aligned with programme aims; relationship to child outcomes less well explained; child / youth outcomes not measured as part of this study | Moderate |
| Lachman et al (2017) | Social support not conceptualised or defined; mechanisms shown in theory of change graph with only very short explanation to it; child outcome measured with standard scales but only one dimension (behaviour) | Low |
| Letourneau et al (2011) | Social support as specific programme aim but limited theory of how to increase social support | Moderate |
| Leventhal et al (2015) | Social support not a primary programme goal and not conceptualised; mechanisms as to how to increase social support and how this leads to improved child outcomes described only briefly; standardised child outcome measures | Low |
| Marcynyszyn et al (2011) | Social support not a primary programme goal but considered an important mediator that is being investigated in this study as a process indicator; very little explanation on how social support is expected to increase and how this would improve child outcomes; both social support and child outcomes measured with standardised tools | Low |
| Mitchell et al (2015) | Social support not explicitly conceptualised; however, it is a primary goal of the programme; mechanisms how to increase social support and how this can lead to improved child development outcomes well conceptualised | Moderate |
| Nabuco et al (2014) | Overall social support is not well conceptualised or defined; social support is not a primary programme goal; mechanisms for how the intervention increases social support and how this leads to improved child development outcomes are explained; standardised social support and child outcome measures | Moderate |
| Pancer et al (2013) | Limited hypothesis and findings in regards to social support; social support and child outcomes measured with standardised scales | Low |
| Parcel and Pennell (2012) | Increasing social support (conceptualised as family and school social capital) is primary programme goal; social support limited to family and school social capital; child outcomes relate to social adjustment, school achievements and mental health (but latter not clear how it should be measured); mechanisms to increase social support and how this leads to improved child outcomes described but without much detail | Moderate |
| Romjinders et al (2017) | Range of evidence of why social support is low in this population; focus on one mechanisms which is acceptance and tolerance but not clear how social support expected to change outside group; no clear programme theory | Moderate |
| Schwartz et al (2013) | Study provides new findings on how social support might lead to improved child outcomes; not clear to what extent this is based on expected mechanisms; most mechanisms refer to how/ why natural mentoring might increase social support but less clear how this was expected to change youth outcomes (i.e. lacks evidence on what are important mechanisms or factors for improving child or youth wellbeing) | Moderate |
| Stubbs and Achat 2016 | Increasing social support was primary programme goal and some explanation and evidence as to how this might be achieved and how this might improve child outcomes; no standardised measure for social support or child outcomes; child wellbeing only captured in terms of process indicators | Moderate |
| Swenson et al (2010) | Social support is an important part of the model, and was shown to improve post-intervention compared to controls, but it did not appear to be a significant part of the intervention as implemented, although this may be also due to reporting issues | Moderate |
| Valdez et al. (2011) | Social support not conceptualised but measured with standardised scale; programme theory does not explain how the intervention is expected to increase social support, and how social support is expected to lead to improved child outcomes; child outcomes measured with standardised scales | Low |
| Valdez et al. (2013) | Social support not conceptualised but measured with standardised scale; programme theory does not explain how the intervention is expected to increase social support, and how social support is expected to lead to improved child outcomes; child outcomes measured with standardised scales | Low |
| Van Dam et al. (2017) | Social support is conceptualised; increasing social support to achieve child outcomes is programme goal and but not much explanation as to how this is achieved (assumption that providing a mentor will lead to this increased support); not much detail on mechanisms to improved child outcomes (and which ones specifically) social support and child outcomes not measured with standardised scales | Moderate |
| Van Voorhees et al. (2008) | Social support is one of several programme goals but has not been conceptualised; some, but not much, information about how to improve social support and how social support improves child outcomes; social support and child outcomes measured with standardised scales | Moderate |
| Vazquez et al. (2017) | Social support measured with a standardised tool but not conceptualised and unclear whether it is considered a primary programme goal; there is also no programme theory as to how social support is expected to improve child outcomes; in terms of child outcomes only child behaviour measured with standardised sub scale; | Low |
| Vella et al. (2018) | Social support increase is underlying programme goal but not conceptualised; mechanisms for increasing social support and achieving child outcomes not well described; Social support and child outcomes measured with standardised scales | Low |

Asghar, K., Mayevskaya, Y., Sommer, M., Razzaque, A., Laird, B., Khan, Y., et al. (2018). Promoting Adolescent Girls' Well-Being in Pakistan: a Mixed-Methods Study of Change Over Time, Feasibility, and Acceptability, of the COMPASS Program. *Prev Sci*.

Ayton, D., & Joss, N. (2016). Empowering vulnerable parents through a family mentoring program. *Aust J Prim Health,* 22, 320-326.

Branch, S., Homel, R., & Freiberg, K. (2013). Making the developmental system work better for children: lessons learned implementing an innovative programme. *Child & Family Social Work,* 18, 294-304.

Byrne, S., Rodrigo, M.J., & Martin, J.C. (2012). Influence of form and timing of social support on parental outcomes of a child-maltreatment prevention program. *Children and Youth Services Review,* 34, 2495-2503.

Cho, Y., Hirose, T., Tomita, N., Shirakawa, S., Murase, K., Komoto, K., et al. (2013). Infant Mental Health Intervention for Preterm Infants in Japan: Promotions of Maternal Mental Health, Mother-Infant Interactions, and Social Support by Providing Continuous Home Visits until the Corrected Infant Age of 12 Months. *Infant Mental Health Journal,* 34, 47-59.

Cluver, L.D., Lachman, J.M., Ward, C.L., Gardner, F., Peterson, T., Hutchings, J.M., et al. (2017). Development of a Parenting Support Program to Prevent Abuse of Adolescents in South Africa: Findings From a Pilot Pre-Post Study. *Research on Social Work Practice,* 27, 758-766.

Deutsch, N.L., Reitz-Krueger, C.L., Henneberger, A.K., Ehrlich, V.A.F., & Lawrence, E.C. (2017). "It Gave Me Ways to Solve Problems and Ways to Talk to People": Outcomes From a Combined Group and One-on-One Mentoring Program for Early Adolescent Girls. *Journal of Adolescent Research,* 32, 291-322.

DeWit, D., DuBois, D., Erdem, G., Larose, S., Lipman, E., DeWit, D.J., et al. (2016). The Role of Program-Supported Mentoring Relationships in Promoting Youth Mental Health, Behavioral and Developmental Outcomes. *Prevention Science,* 17, 646-657.

Doty, J.L., Davis, L., & Arditti, J.A. (2017). Cascading Resilience: Leverage Points in Promoting Parent and Child Well-Being. *Journal of Family Theory & Review,* 9, 111-126.

Drummond, J., Schnirer, L., So, S., Mayan, M., Williamson, D.L., Bisanz, J., et al. (2014). The protocol for the Families First Edmonton trial (FFE): a randomized community-based trial to compare four service integration approaches for families with low-income. *BMC Health Serv Res,* 14, 223.

Eddy, J., Martinez, C., Grossman, J., Cearley, J., Herrera, D., Wheeler, A., et al. (2017). A Randomized Controlled Trial of a Long-Term Professional Mentoring Program for Children at Risk: Outcomes Across the First 5 Years. *Prevention Science,* 18, 899-910.

Hauken, M.A., Senneseth, M., Dyregrov, A., & Dyregrov, K. (2015). Optimizing Social Network Support to Families Living With Parental Cancer: Research Protocol for the Cancer-PEPSONE Study. *JMIR Res Protoc,* 4, e142.

Ingram, S.D., Cash, S.J., Oats, R.G., Simpson, A., & Thompson, R.W. (2015). Development of an evidence-informed in-home family services model for families and children at risk of abuse and neglect. *Child & Family Social Work,* 20, 139-148.

January, S.-A., Duppong Hurley, K., Stevens, A., Kutash, K., Duchnowski, A., & Pereda, N. (2016). Evaluation of a Community-Based Peer-to-Peer Support Program for Parents of At-Risk Youth with Emotional and Behavioral Difficulties. *Journal of Child & Family Studies,* 25, 836-844.

Lachman, J.M., Cluver, L., Ward, C.L., Hutchings, J., Mlotshwa, S., Wessels, I., et al. (2017). Randomized controlled trial of a parenting program to reduce the risk of child maltreatment in South Africa. *Child Abuse Negl,* 72, 338-351.

Letourneau, N., Stewart, M., Dennis, C.L., Hegadoren, K., Duffett-Leger, L., & Watson, B. (2011). Effect of home-based peer support on maternal-infant interactions among women with postpartum depression: a randomized, controlled trial. *Int J Ment Health Nurs,* 20, 345-357.

Leventhal, K.S., Gillham, J., DeMaria, L., Andrew, G., Peabody, J., & Leventhal, S. (2015). Building psychosocial assets and wellbeing among adolescent girls: A randomized controlled trial. *J Adolesc,* 45, 284-295.

Marcynyszyn, L.A., Maher, E.J., & Corwin, T.W. (2011). Getting with the (evidence-based) program: An evaluation of the Incredible Years Parenting Training Program in child welfare. *Children & Youth Services Review,* 33, 747-757.

Mitchell, G., Absler, D., & Humphreys, C. (2015). "She's just like me": The Role of the Mentor with Vulnerable Mothers and their Infants. *Children Australia,* 40, 33-42.

Nabuco, M.E., Aguiar, M.S., Costa, C., & Morais, D. (2014). Evaluation of the effectiveness of the implementation of the A PAR parental intervention programme in Portugal. Child development and parenting support. *European Early Childhood Education Research Journal,* 22, 554-572.

Pancer, S.M., Nelson, G., Hasford, J., & Loomis, C. (2013). The Better Beginnings, Better Futures Project: Long-term Parent, Family, and Community Outcomes of a Universal, Comprehensive, Community-Based Prevention Approach for Primary School Children and their Families. *Journal of Community & Applied Social Psychology,* 23, 187-205.

Parcel, T.L., & Pennell, J. (2012). Child and Family Teams Building Social Capital for At-Risk Students: A Research Note. pp. 75-91).

Romijnders, K.A., Wilkerson, J.M., Crutzen, R., Kok, G., Bauldry, J., & Lawler, S.M. (2017). Strengthening Social Ties to Increase Confidence and Self-Esteem Among Sexual and Gender Minority Youth. *Health Promot Pract,* 18, 341-347.

Schwartz, S., Rhodes, J., Spencer, R., & Grossman, J. (2013). Youth Initiated Mentoring: Investigating a New Approach to Working with Vulnerable Adolescents. *American Journal of Community Psychology,* 52, 155-169.

Stubbs, J.M., & Achat, H.M. (2016). Sustained health home visiting can improve families' social support and community connectedness. *Contemp Nurse,* 52, 286-299.

Swenson, C.C., Schaeffer, C.M., Henggeler, S.W., Faldowski, R., & Mayhew, A.M. (2010). Multisystemic Therapy for Child Abuse and Neglect: a randomized effectiveness trial. *J Fam Psychol,* 24, 497-507.

Valdez, C.R., Mills, C.L., Barrueco, S., Leis, J., & Riley, A.W. (2011). A Pilot Study of a Family-Focused Intervention for Children and Families Affected by Maternal Depression. *J Fam Ther,* 33, 3-19.

Valdez, C.R., Padilla, B., Moore, S.M., & Magana, S. (2013). Feasibility, acceptability, and preliminary outcomes of the Fortalezas Familiares intervention for latino families facing maternal depression. *Fam Process,* 52, 394-410.

van Dam, L., Neels, S., de Winter, M., Branje, S., Wijsbroek, S., Hutschemaekers, G., et al. (2017). Youth Initiated Mentors: Do They Offer an Alternative for Out-of-Home Placement in Youth Care? *British Journal of Social Work,* 47, 1764-1780.

Van Voorhees, B.W., Vanderplough-Booth, K., Fogel, J., Gladstone, T., Bell, C., Stuart, S., et al. (2008). Integrative internet-based depression prevention for adolescents: a randomized clinical trial in primary care for vulnerability and protective factors. *J Can Acad Child Adolesc Psychiatry,* 17, 184-196.

Vazquez, N., Molina, M.C., Ramos, P., & Artazcoz, L. (2017). Effectiveness of a parent-training program in Spain: reducing the Southern European evaluation gap. *Gac Sanit*.

Vella, S.A., Swann, C., Batterham, M., Boydell, K.M., Eckermann, S., Fogarty, A., et al. (2018). Ahead of the game protocol: a multi-component, community sport-based program targeting prevention, promotion and early intervention for mental health among adolescent males. *BMC Public Health,* 18, 390.
