# Supplementary material for: Mobilising social support to improve mental health for children and adolescents: A systematic review using principles of realist synthesis
Source: PLoS One. 2021 May 20;16(5):e0251750. doi: 10.1371/journal.pone.0251750 (PMC8136658; doi:10.1371/journal.pone.0251750)
Supplement: S2 Table — (DOCX) [file pone.0251750.s003.docx]

Table S2: Assessment of quality of included studies

| Quantitative randomised controlled trials | | | | | | | | | | | |
| --- | --- | --- | --- | --- | --- | --- | --- | --- | --- | --- | --- |
| Study ID | Randomisation appropriately performed | | Groups comparable at baseline | | Complete outcomes data | | Outcome assessor blinded | | Adherence to intervention | | Summary rating |
| Drummond et al (2014) | Yes | | Not yet known | | Not yet known | | Yes | | Not yet known | | High (expected based on study protocol) |
| Eddy et al (2017) | No | | Can’t tell | | No | | No | | No | | Low |
| Hauken et al (2015) | No | | Not yet known | | Not yet known | | No | | Not yet known | | Moderate (expected based on study protocol) |
| Lachman et al (2017) | Yes | | Yes | | Yes | | Yes | | Yes | | High |
| Letourneau et al (2011) | No | | Yes | | No | | Yes | | No | | Moderate |
| Leventhal et al (2015) | Yes | | Yes | | Yes | | No | | Yes | | High |
| Swenson et al (2010) | Yes | | Yes | | Yes | | No | | Yes | | High |
| Van Voorhees et al (2008) | Yes | | Yes | | Yes | | No | | Yes | | High |
| Quantitative non-randomised studies | | | | | | | | | | | |
|  | Participants representative of target population | | Measurements appropriate regarding both the outcome and the intervention/ exposure | | Outcomes data complete | | Confounders accounted for in analysis | | Intervention administered as intended | | Summary rating |
| Asghar et al (2018) | Can’t tell | | No | | No | | No | | No | | Low |
| Bohleber et al (2016) | No | | Yes | | No | | Yes | | No | | Low |
| Byrne et al (2012) | No | | Yes | | Can’t tell | | Yes | | Yes | | Moderate |
| Cho et al (2013) | No | | Yes | | No | | No | | Yes | | Moderate |
| Cluver et al (2017) | Yes | | No | | Yes | | No | | Yes | | Moderate |
| DeWit et al (2016) | Yes | | Yes | | No | | Yes | | Can’t tell | | Moderate |
| January et al (2016) | Can’t tell | | No | | Can’t tell | | Yes | | Yes | | Low |
| Marcynyszyn et al (2011) | Yes | | Yes | | Yes | | No | | No | | Moderate |
| Nabuco et al (2014) | Can’t tell | | Can’t tell | | Can’t tell | | Yes | | Yes | | Moderate |
| Pancer et al (2013) | Can’t tell | | Yes | | Yes | | Yes | | Yes | | High |
| Schwartz et al (2013) | Yes | | No | | Yes | | Yes | | Can’t tell | | Moderate |
| Stubbs and Achat (2016) | No | | No | | Can’t tell | | No | | No | | Low |
| Valdez et al (2011) | Can’t tell | | No | | No | | No | | No | | Low |
| Valdez et al (2013) | Can’t tell | | Yes | | No | | No | | Yes | | Moderate |
| Van Dam et al (2017) | Yes | | No | | Yes | | No | | No | | Moderate |
| Vazquez et al (2017) | Can’t tell | | Can’t tell | | No | | No | | Can’t tell | | Low |
| Vella et al (2018) | Can’t tell | | Yes | | Not yet known | | Yes | | Not yet known | | Moderate (expected based on study protocol) |
| Qualitative studies | | | | | | | | | | | |
| Study ID | Appropriate qualitative approach | | Adequate data collection methods | | Findings adequately derived from data | | Interpretation of results sufficiently substantiated by data | | Coherence between data sources, collection, analysis and interpretation | | Summary rating |
|  |  | |  | |  | |  | |  | |  |
| Asghar et al (2018) | Yes | | Yes | | Yes | | No | | No | | Moderate |
| Ayton and Joss (2014) | Yes | | No | | Yes | | Yes | | Yes | | High |
| Branch et al (2013) | Yes | | No | | Can’t tell | | No | | No | | Low |
| Deutsch et al (2017) | Yes | | Yes | | Yes | | Yes | | Yes | | High |
| Mitchell et al (2015) | Can’t tell | | No | | Can’t tell | | Can’t tell | | Can’t tell | | Low |
| Romjinders et al (2017) | Yes | | Yes | | Yes | | Yes | | Yes | | High |
| Schwartz et al 2013 | Yes | | Yes | | Yes | | Yes | | Yes | | High |
| Vazquez et al (2017) | Yes | | Can’t tell | | Can’t tell | | Can’t tell | | Can’t tell | | Low |
| Mixed methods studies | | | | | | | | | | | |
|  | Adequate rationale for using mixed method | Different components effectively integrated | | Outputs of the integration of qual. and quant. components adequately interpreted | | Divergences and inconsistencies between quant. And qual. results adequately addressed | | Different components adhere to quality criteria of each tradition of methods involved | | Summary score | |
| Asghar et al (2018) | No | Yes | | No | | Yes | | No | | Moderate | |
| Schwartz et al (2013) | Yes | Yes | | Yes | | Yes | | No | | High | |
