# Supplementary material for: Mobilising social support to improve mental health for children and adolescents: A systematic review using principles of realist synthesis
Source: PLoS One. 2021 May 20;16(5):e0251750. doi: 10.1371/journal.pone.0251750 (PMC8136658; doi:10.1371/journal.pone.0251750)
Supplement: S3 Table — (DOCX) [file pone.0251750.s004.docx]

Table S3: Characteristics of included studies concerned with infants (0 to 2 years)

| Study details | Intervention characteristics | Social support aim(s) and measure(s) | Child outcome measure(s) |
| --- | --- | --- | --- |
| Cho et al (2013)  Type: Evaluation (non-randomised control group design)  Size: N=66 (intervention group: n=26; control group: n=40)  Setting(s): Hospital; 3 hospitals with Growing Care Units, Tokyo  Country: Japan  Relevance: Low  Quality: Moderate | Hospital and home visiting program ‘The Japanese Infant Mental Health Program’ (JIMHP); JIMHP helpers meet with mother once in hospital and 5 times at home  Delivered by:  JIMH helpers (clinicians) trained by European Early Promotion Project; 10 workshops (including role play e.g. active listening)  Delivered to:  Mothers of infants (pre-term - until 12 months of corrected age) | To promote mothers’ social support (mainly informational support from relationships with healthcare professionals)  Support from partner, family, friends, health professionals measured with modified version of Social Support Scale | Postural-motor, cognitive-adaptive, and language-social skills measured with standardized child development test |
| Letourneau et al (2011)  Type: Evaluation (RCT)  Size: N=60 (intervention group: n=27; control group: n=33)  Setting(s): Community; recruited via campaign and health professionals in two Canadian provinces (Alberta and New Brunswick)  Country: Canada  Relevance: Moderate  Quality: Moderate | Home-based peer support; included maternal–infant interaction teaching (Keys to Caregiving programme); 12 weeks  Delivered by:  Volunteers (mothers recovered from postpartum depression); 8-hour classroom-based sessions and regular follow-up, debriefing  Delivered to:  Mothers with depression of infants (<9 months) | To increase maternal social support, including her perceptions thereof  Perceived support (guidance, reliable alliance, reassurance of worth, attachment, social integration, opportunity for nurturance) measured with Social Provisions Scale | Maternal reports of social–emotional development measured with Infant Characteristics Questionnaire  Stress measured with cortisol levels |
| Mitchell et al (2015)  Type: Evaluation (qualitative)  Size: N=20  Setting(s): Public hospital specialist maternity service; north east region of Melbourne  Country: Australia  Relevance: Moderate  Quality: Low | Community-based pilot program of volunteer home visiting (‘Mentoring Mums’); volunteers walks alongside isolated mother from late pregnancy/ early infancy for period of up to 2 years  Delivered by:  Community volunteers (mothers); 3-days training program and ongoing sessions; support from coordinator and caseworker, who also works with mother  Delivered to:  Mothers of infants | To help mothers engage with local services and supports by modelling friendliness and providing opportunities for practicing social skills  Changes in social isolation measured in form of changes in access to services and interaction with formal (health) service systems, and the local community (various data sources) | Child development across a range of milestones; increased mother-infant attachment (various data sources) |
| Stubbs and Achat (2016)  Type: Evaluation (pre post design)  Size: N=118 (baseline), N=65 (follow up)  Setting(s): Community health centre in western Sydney, North South Wales (NSW)  Country: Australia  Relevance: Moderate  Quality: Moderate | ‘Family Partnership Model’; delivered through home visits; average 2.2 direct contacts per month (median 60 minutes); duration min. 6 months  Delivered by:  Child and family health nurses trained in Family Partnership Model  Delivered to:  Parents of infants (median age 7 weeks) | To link isolated families to services and enhancing community and support networks, and offer social support  Parents’ perceived support from interpersonal relationships; given + received formal and informal support (various questions + data sources) | Questions whether/ how child development was impacted by program |
