# Supplementary material for: Mobilising social support to improve mental health for children and adolescents: A systematic review using principles of realist synthesis
Source: PLoS One. 2021 May 20;16(5):e0251750. doi: 10.1371/journal.pone.0251750 (PMC8136658; doi:10.1371/journal.pone.0251750)
Supplement: S4 Table — (DOCX) [file pone.0251750.s005.docx]

Table S4: Characteristics of included studies concerned with children (3 to 9 years)

| Study details | Intervention characteristics | Social support measure | Child outcome measure(s) |
| --- | --- | --- | --- |
| Ayton & Joss (2016)  Type: Evaluation (qualitative)  Size: Mentors n=27; Mentees n=12  Setting(s): Community; two church sites, Mornington Peninsula, Victoria  Country: Australia  Relevance: Low  Quality: High | Home-visiting mentoring program (‘Creating Opportunities and Casting Hope’; COACH) for vulnerable parents; duration 12 months  Delivered by:  Volunteers (church attendees) interviewed and trained by program staff who are social workers (14 hours); plus mentor training manual  Delivered to:  Parents of children < 12 years | To help parent to reach own goals related to social support such as developing relationships with others and establishing community connections  Parent’s perceived benefits of mentoring support received; goals achieved in regards to social support goal | Questions about health and wellbeing of children |
| Branch et al (2013)  Type: Evaluation (qualitative)  Sample: Semi-structured interviews (n=22) with parents, teachers, program staff; survey (n=20) with parents and teachers  Setting(s): Residential sites (no details provided)  Country: Australia  Relevance: Low  Quality: Low | Circles of Care; involves regular meetings where all members of the Circle participate (ideally once per school term)  Delivered by:  Community workers, which are trained, coached and supervised by a coordinator  Delivered to:  Children (5 to 11 years), children’s family, teachers, community workers, others | To enhance support networks around child and provide access to formal social support, including through school integration  Questions about parents’ support from school, community welfare services, and their participation; support and assistance received by child | Questions about improvements in child behaviour |
| Byrne et al 2012  Type: Evaluation (pre post design)  Sample: N=494  Setting(s): Community; family program in regions of Castile and Leon  Country: Spain  Relevance: Moderate  Quality: Moderate | Parenting programme ‘The Personal Family and Family Support program’ Apoyo Personal y Familiar (APF); 90 min session per week for 8 months; plus two warm up sessions at start of program; group based  Delivered by:  Facilitators (locally trained social workers; trained 25 hours before program; additional training/ supervision session half way through program)  Delivered to:  Parents of children (age not specified) | To reinforce parents’ perception of and increase satisfaction with social support networks  Various sources of informal and formal social support measured with modified Scale of Social Support in Informal and Formal systems and Social Support Questionnaire | Parents’ beliefs around child development measured with questionnaire  Various aspects of child rearing, parental agency (measured with standardised scales) |
| Doty et al (2017)  Study type: Conceptual (programme theory development)  Sample: Not applicable  Setting(s): Not applicable  Country: USA  Relevance: Moderate  Quality: Not applicable | Holistic parenting interventions focused on strengthening parent-child relationship and building family social capital  Delivered by:  Practitioners (not specified)  Delivered to:  Parents of children (age not specified) | To help parents build social networks by increasing their confidence to mobilise support and build social capital for child  Social support conceptualised as long-term social resources available to family | Expected improvements in child development, wellbeing and resilience |
| Drummond et al. (2014)  Type: Evaluation (protocol; RCT)  Sample: N=1,173 (n=291 to 293 in each of three intervention groups; control group: n=291)  Setting(s): Community; City of Edmonton; recruitment via campaign and welfare programs  Country: Canada  Relevance: Moderate  Quality: High | Families First Edmonton (FFE), service integration approaches for low income families; 1.4 to 4.6 hours per month; duration 18 to 24 months  FFE described as complex programs provided from multi-level and sectoral collaborations; incorporate principles of family-centredness, cultural sensitivity, capacity building and reflection  Delivered by:  Families Matter partnership of four pre-existing community services  Delivered to:  Families with at least one child <12 years (study protocol , no mean age) | To increase formal social support of families, including through service integration (e.g. school and childcare)  Family linkage to services and resources measured with Family Services Inventory (FSI)  Social functions and provisions from relationships with others measured with Social Provision Scale (SPS) | Child psychosocial health measured with Behavioral Assessment System for Children (BASC) |
| Eddy et al (2017)  Type: Evaluation (multi-site RCT; growth curve analysis over 5 years)  Sample: N=278 children, and one of their caregivers; Year 5: N=184 (intervention group: N=109; control group: N=75)  Setting(s): Public elementary schools in Boston, New York City, Portland (Oregon), Seattle  Country: USA  Relevance: Moderate  Quality: Low | Paid professional mentoring program ‘Friends of the Children’ (FOTC); average 4 hours of mentoring per week (plus additional activities); average duration 4.4 years; one-to-one and group activities  Delivered by:  Mentors (organised through non-profit community-based organisations); 1-week training and ongoing supervision by FOTC mentors as well as FOTC programme director and external consultants  Delivered to:  Children (mean age 6.5 years) | To provide a child with social support opportunities; including access to material and practical support (e.g. health care, schooling)  Mentors asked about amount of contact and specific activities they had with or for child | Scales from Child Behaviour Checklist (CBCL)  Behaviour and Emotional Rating Scale (BERS-2)  Child reported antisocial behaviour scale and deviant peers scale |
| Ingram et al (2015)  Type: Conceptual (description of programme theory)  Sample: Not applicable  Setting: Boys Town (child welfare treatment provider), District of Columbia  Country: USA  Relevance: Moderate  Quality: Not applicable | Type of intervention:  In-home family service model; family goals established and plans put in place to meet those; Social Network Map used to formalise initial assessment and assist with service planning; on average n=105 days per family  Delivered by:  Caseworkers with access to web-based integrated assessment and service system  Delivered to:  Parents of children (age not specified) | To increase informal and formal supports for families that help them maintain progress after formal services ended; by teaching them skills to ask for and utilise social support  Various formal and informal social support perceived and received by family measured with Social Network Map | Child behaviour, wellbeing  Perceived levels of stress related to social support system (e.g. Strengths and Stressors tool) |
| Lachman et al. (2017)  Type: RCT  Sample: N=68 (intervention group: n=34; control group: n=34)  Setting(s): Community; Khayelitsha (suburban region of Cape town)  Country: South Africa  Relevance: Low  Quality: High | Parent training program (Sinovuyo Caring Families Program); 12 weekly session; each 2-3 hours; 10-14 parents per group; home consultations when parents miss session  Delivered by:  Community-based facilitators trained by program staff  Delivered to:  Parents of children aged 3 to 8 years | To improve parent’s social support  Parent’s perceived social support (various sources) measured with Multidimensional Scale of Perceived Social Support (MSPsocial support) | Child behaviour measured through Sinovuyo Observational  Coding System (SOCS) and Eyberg Child Behavior Inventory (ECBI) |
| Marcynyszyn et al (2011)  Type: Evaluation (pre post)  Sample: N=41  Setting(s): Two child welfare agencies (Casey Family Program), New York State  Country: USA  Relevance: Low  Quality: Moderate | Group-based parent training and education program ‘Incredible Years’ (IY); 12 to 14 weeks duration; 2 hours each week  Delivered by:  Group leaders who are therapists; trained in 2 day workshop by IY program developers; clinical supervision  Delivered to:  Parents of children aged between 6 months and 8 years | To improve perceived social support of parents  Parents’ perceived social support (various sources) measured with Multidimensional Scale of Perceived Social Support (MSPsocial support) and Family Support Scale (Fsocial support) | Child behaviour measured in previous trials (improvements well established) |
| Nabuco et al (2014)  Type: Evaluation (cross sectional, quasi-experimental)  Sample: 2008: N=103 (intervention group: N=57, control group=46); 2009: N=142 (intervention group: N=71, control group: N=71); 2010: N=187 (intervention group=92, control group=95)  Setting(s): Community; A-PAR program setting and recruitment from crèches and nurseries; Greater Lisbon  Country: Portugal  Relevance: Moderate  Quality: Moderate | Early childhood education and parenting support (A-PAR); based on UK’s Parents Early Education Partnership (PEEP); one-hour weekly group sessions with parents (or significant other) and child; provided for one year  Delivered by:  Leaders with four year university degree in early childhood education; trained 25 hours by program developer; ongoing training (2 hours per month) supervision by program staff  Delivered to:  Parents of children (up to 6 years) | To convince parents of benefit of social support for child welfare and to promote their social support networks  Parents’ social support measured with Social Support Questionnaire (QAS) | Socio-emotional development measured with Social Competence Scale (QCS) and Emotional Activity and Sociability Scale (EEAS)  Cognitive development measured with Wechsler Preschool and Primary Scale of Intelligence-Revised (WPPSI) |
| Pancer et al (2013)  Type: Evaluation (cohort design; follow up when youth were 18 to 19 years)  Sample: N=626 (intervention group: N=401; control group: N=225)  Setting(s): Community; schools in three regions; Ontario  Country: Canada  Relevance: Low  Quality: High | Better Beginnings, Better Future (BBBF); community-driven programmes offering wide range of education, play, sports, arts or leisure activities  Delivered by:  Various members of the community, parents, children  Delivered to:  Parents and their children (4 to 8 years) | To help parents gain access to social and tangible support  Parents’ social support measured with Social Provisions Scale  Other aspects of parents’ perceived social support measured with single questions | Children’s social behaviours measured on subscales from the Social Skills Rating Scale  Cognitive and academic performance (various measures) |
| Parcel and Pennell (2012)  Type: Conceptual (programme theory development)  Sample: Not applicable  Setting(s): Community; schools  Country: USA  Relevance: Moderate  Quality: Not applicable | Child and family teams (CFTs) - teams around child to plan how to support child and family  Delivered by:  CFT facilitators and other involved school staff  Delivered to:  Children (school age), (extended) family, school teachers | To wrap a comprehensive and unified array of services and supports around children and their families | Child development, mental health and wellbeing  Social adjustment and behaviour; crimes and acting out |
| Vazquez et al. (2017)  Type: Evaluation (pre post)  Sample: N=216 (baseline); N=130 (follow up)  Setting(s): Community; various recruitment strategies; Barcelona  Country: Spain  Relevance: Low  Quality: Low | Parenting Skills Program for families (PSP); 11 weekly sessions of 90 minutes; groups of 10-14 parents (children participate in two sessions)  Delivered by:  Practitioners in community services; trained in PSP  Delivered to:  Parents with children (aged 2 to 12 years; most children between 3 and 5) | To increase parents’ perceived social support  Parents’ perceived social support (confident and emotional) measured with Duke-UNC Functional Social Support Questionnaire  Open questions about social support to parent | Child behaviour through sub-scale of Strength and Difficulties Questionnaire  Children’s emotional self-regulation through single questions  Children behaviour effects through open questions |
