# Supplementary material for: Mobilising social support to improve mental health for children and adolescents: A systematic review using principles of realist synthesis
Source: PLoS One. 2021 May 20;16(5):e0251750. doi: 10.1371/journal.pone.0251750 (PMC8136658; doi:10.1371/journal.pone.0251750)
Supplement: S5 Table — (DOCX) [file pone.0251750.s006.docx]

Table S5: Characteristics of included studies concerned with adolescents (10 to 18 years)

| Study details | Intervention characteristics | Social support aim(s) and measure(s) | Child outcome measure(s) |
| --- | --- | --- | --- |
| Asghar et al. (2018)  Type: Evaluation (mixed method)  Sample: Quant.: N=78; Qual.: N=15  Setting(s): Community and camps; Women Community Centres (n=9); four located in camps for displaced people; different districts of Khyber-Pakhtunkwa region  Country: Pakistan  Relevance: Low  Quality: Moderate | Creating Opportunities through Mentorship, Parental Involvement, and Safe Spaces (COMPAsocial support) program; weekly 45 to 60 minutes group sessions plus 30 mins unstructured time; 26 sessions for girls; 14 sessions for female caregivers  Delivered by:  Mentors employed by implementing organisations, which received targeted training and ongoing support from program developers  Delivered to:  Adolescent girls (12 to 19 years) and their female caregivers | To increase girls’ social assets over time (including friends and trusted adults)  Quality of relationship with caregivers measured in comfort talking about intimate topics: education, earning a living, marriage, puberty | Self-esteem measured with Rosenberg Self-esteem Scale  Hope measured with Children's Hope Scale  Self-reported school attendance |
| Bohleber et al. (2016)  Type: Evaluation (non-randomised control group design)  Sample: 1^st^ sample youth in employment: intervention group: N=546; control group: N=395; 2^nd^ sample of youth in unemployment: intervention group: N=73; control group: N=120  Setting(s): Swiss company and public employment transition program  Country: Switzerland | Mental health promotion app (‘Companion App’), which provides peer mentoring system, group discussions, links to websites on mental health issues and leisure activities information, anonymous professional counselling service; provided over 10 months  Development in consultation with youth focus group and users have opportunity to provide feedback and suggest improvements  Delivered by:  Peer mentors (no further details provided on how they were trained); research team developed and maintained the App  Delivered to:  Young people (mean age 17 years) | To increase adolescents’ perceived social support; to enhance their feelings of connectedness to school and parents  Satisfaction with social support and reciprocity in social support measured with two scales of Social Support Questionnaire | *Chronic stress measured with Trier Inventory of Chronic Stress screening scale (TICS-SCsocial support)* |
| Cluver et al (2017)  Type: Evaluation (pre post; pilot)  Sample: N=60  Setting(s): Community; community-based organisation in rural area of Eastern Cape province  Country: South Africa  Relevance: Low  Quality: Moderate | Parenting program ‘Sinovuyo Caring Families Teen Programme’ (Sinovuyo = we have happiness or joy); 10-sessions  Delivered by:  Community staff with experience of conducting parenting programmes; trained 1 week on collaborative learning techniques, modelling praise and problem solving skills  Delivered to:  Children (aged 10 to 17 years) and their mothers or female caregivers | To improve parent and adolescent social support  Parents’ and youth’s social support (emotional, tangible, affectionate) and positive social interaction measured with the Medical Outcome Study Social Support Survey | Adolescent behaviour problems measured using subscales of the Child Behaviour Checklist (CBC) |
| Deutsch et al (2017)  Type: Evaluation (qualitative)  Sample: N=113  Setting(s): Community; schools  Country: USA  Relevance: Moderate  Quality: High | Youth mentoring programme (Young Women Leaders Program, YWLP); one-to-one and group component; frequency or duration not specified  Delivered by:  Women mentors (volunteers); training (amount, duration not specified)  Delivered to:  Children (mean age 12) | To optimise social support  Qualitative exploration of expanded peer networks through making new friends as well as becoming closer to people they already knew | Youth development (social, emotional, cognitive, identity)  Qualitative exploration of self-regulation |
| DeWit et al (2016)  Type: Evaluation (cohort; 18 months follow up)  Sample: N=859  Setting(s): Community; 21 Big Brothers Big Sisters agencies across Canada  Country: Canada  Relevance: Moderate  Quality: Moderate | Youth mentoring ‘Big Brothers Big Sisters’ community mentoring relationship’ (BBBS); 2 to 4 hours per week for period of one year  Delivered by:  Mentors (volunteers) and caseworkers trained by programme staff  Delivered to:  Adolescents (up to 17 years) | To strengthen, nurture and support relationships of young persons with caring and responsible adults  Perceived peer and teacher support via subscales of the Social Support Appraisal Scale (SSAS)  Perceived parent emotional support measured with sub scale of the Wills Parental Support Scale (WPSS) | Various mental health problems measured wit Strengths and Difficulties Questionnaire (SDQ)  Depression, anxiety, stress measured with: Generalized Social Anxiety and Distress sub-scale (SAD-G) of Revised Social Anxiety Scale for Children (SASC-R), subscale from Center for Epidemiology Studies Depression Scale (CES-DC) |
| Hauken et al. (2015)  Type: Evaluation (protocol; RCT)  Sample: Planned: N=120 parents (n=60); children (n=60); equal allocation between IG and CG  Setting(s): Community; national recruitment strategy including cancer charities, wider range of health professionals  Country: Norway  Relevance: High  Quality: Moderate | Psycho-educational program for the social network (‘PEPSONE’) approx. 3 hours session at families’ home  Delivered by:  Clinical psychologists  Delivered to:  Family with child aged 8-18 years; social network members (study protocol, no mean age) | To optimize social network support of families whose children live with cancer  Parents’ social support after crisis measured with the Crisis Support Scale (CSS)  Parents and social network members received/ provided social support measured with the Assistance Questionnaire-Receivers / Providers of support (AQR/ AQP) | Anxiety measured with Revised Child Manifest Anxiety Scale (RCMAS)  Quality of life measured with the Kinder Lebensqualität (KINDL) |
| January et al (2016)  Type: Evaluation (pre post design)  Sample: N=139  Setting(s): Community organisation in Western United States  Country: USA  Relevance: Moderate  Quality: Low | Peer-to-peer support prevention program delivered via telephone (Parent Connectors); Family contact log completed, reviewed and updated by Parent Connector; duration: 3 months  Delivered by:  Volunteers; trained 3 days through a manualized training curriculum and weekly 2 hours supervision sessions by trained mental health practitioner  Delivered to:  Parents of children (mean age 11 years) | To promote positive attitudes toward building social support networks; to provide emotional support to reduce feelings of blame and stigma; instrumental support to reduce basic needs such as clothing, food; informational support to increase academic and behavioral success  Perceived informal support from others and perceived access to services and tangible goods to assist the family in coping with stress measured with social and concrete support domains of the Protective Factors Survey (PFS) | Children outcomes not reported in this study but previous evaluation of the same intervention provided to youth measured mental health with the Strengths and Difficulties Questionnaire (SDQ) and Brief Impairment Scale (BIS) |
| Leventhal et al. (2015)  Type: RCT (evaluation)  Sample: At follow-up N=2,387 (intervention group: n=1,681; control group: n=706)  Setting(s): Community; Government schools (n=57), Bihar  Country: India  Relevance: Low  Quality: High | Resilience-framework-based intervention (Girls First Resilience Curriculum; RC); over the course of 5 months; 23 weekly facilitated peer-support sessions over 5 months; group based (12-15 girls)  Delivered by:  Local women with at least 10^th^ grade education as group facilitators; 5 days initial and 3 days follow up training by Master Trainers; supervision and refresher training  Delivered to:  Girls (mean age 13 years) | To change perception of benefit of helping each other, social skills to do so and to increase links with peers and community  Extent to which child/ adolescent feels supported and accepted by peers and friends measured with the Social Support and Peers subscales of Kidscreen-52 | Depression with Patient Health Questionnaire-9 (PHQ-9)  Anxiety with General Anxiety Disorder-7 (GAD-7)  Positive psychological sub scale of KIDSCREEN-52  Social-emotional assets with items from the Child and Youth Resilience Measure |
| Romjinders et al (2017)  Study type: Evaluation (qualitative)  Sample: N=12  Setting: Community; drop in program, Houston, Texas  Country: USA  Relevance: Moderate  Quality: High | Drop-in program for sexual and gender minority (SGM) youth (Hatch Youth); drop-in meetings (3 hours, 3 nights a week), youth-led peer support group & mentoring; average duration: 14.5 months  Delivered by:  Volunteers (or sometimes staff) who are trained as group facilitators; youth themselves  Delivered to:  Youth (mean age 18 years) | To increase social support from primary and secondary social ties  Explored qualitatively (emerged as dominant theme during interviews) | Youth confidence and self-esteem explored qualitatively |
| Schwartz et al (2013)  Type: Evaluation (mixed methods)  Sample: Quant. N=1,173; qual. N=30  Setting(s): National Guard Youth Challenge Program sites (n=10) across the country  Country: USA  Relevance: Moderate  Quality: High | Youth initiated mentoring (YIM); weekly contacts between mentor and mentees; at least two face-per-face meetings per month; formal participation: 1 year  Delivered by:  Volunteers (natural mentors), interviewed and trained by program staff  Delivered to:  Youth (16 to 18 years) | To optimise role of social support through caring adult  Different aspects of social support explored qualitatively  Mentoring relationships (frequency, duration and type of contact) through survey questions | Youth educational, vocational, and behavioural outcomes explored qualitatively |
| Swenson et al. (2010)  Type: Evaluation (RCT)  Sample: N=86  Setting(s): Community; Child Protection Services, Charleston County  Country: USA  Relevance: Moderate  Quality: High | ‘Multi-systemic Therapy (MST) – daily individual sessions; duration min. 6 months; 24/7 availability for crises management  Delivered by:  Therapists including MST-trained psychiatrist; MST supervisor  Delivered to:  Families with children 10 to 17 years (mean age 14 years) | To increase (and optimise) social support  Parents’ social support in categories i.e. perceived, appraisal and belonging measured with Interpersonal Support Evaluation List (ISEL) | Youth behavioral and emotional functioning with Child Behavior Checklist (CBCL)  Trauma Symptom Checklist for Children (TSCC)  Parent completed Social Skills Rating System |
| Valdez et al. (2011)  Type: Evaluation (pre post; non-experimental; pilot)  Sample: N=10 mothers; N=16 children  Setting(s): Community; two outpatient mental health clinics, Greater Baltimore, Maryland  Country: USA  Relevance: Low  Quality: Low | Keeping Families Strong (KFS) for families in which mothers has depression; 10 multi-family group sessions  Delivered by:  Adult and children’s mental health clinicians (supervised by research clinicians)  Delivered to:  Mothers in treatment for depression, their partners and children (aged between 9 and 16 years) | To increase interfamily social support  Mothers’ perceived social support (various sources) measured with Multidimensional Scale of Perceived Social Support  Quality of mothers’ intimate relationships measured with Dyadic Adjustment Scale | Behaviour Assessment System for Children (BASC)  Social emotional competence, resilience and coping measured with Child Coping Strategies Checklist (CCSC)  Coping Efficacy Scale |
| Valdez et al. (2013)  ype: Evaluation (pre post; feasibility)  Sample: N=13 families  Setting(s): Community; three community outpatient clinics located in Latino neighbourhoods; middle sized city in the Midwest  Country: USA  Relevance: Low  Quality: Low | Fortalezas Familiares (Family Strengths); adapted from Keeping Families Strong (KSF); 12-week multi-family intervention plus two booster meetings  Delivered by:  Research clinicians in a community agency  Delivered to:  Mothers in treatment for depression, other caregivers and children (aged 9 to 18 years) | To increase interfamily social support  Mothers’ perceived social support (various sources) measured with Multidimensional Scale of Perceived Social Support | Child psychological functioning measured with the Strengths and Difficulties Questionnaire (SDQ)  Child coping measured with subscales of the Children’s Coping Strategies Checklist Revision 1 (CCSC) and Coping Efficacy Scale (CES) |
| Van Dam et al. (2017)  Type: Evaluation (case analysis; cross sectional study)  Sample: N=200 (intervention group: n=96; control group: n=104)  Setting: Residential; youth organisations providing residential care  Country: Netherlands  Relevance: Moderate  Quality: Moderate | ‘Youth Initiated Mentoring’ (YIM); various meetings take place between YIM, young person, family and professionals; duration between 6 and 9 months  Delivered by:  Volunteers (natural mentors)  Delivered to:  Youth (12 to 23 years) | To increase youth collaboration with the family and its social network; to help youth request and maintain support from others  Received social support (emotional, practical, guidance and advice) measured through data records | Youth health and mental wellbeing measured with CAP-J  Dutch classification instrument for youth mental health |
| Van Voorhees et al. (2008)  Type: Evaluation (RCT)  Sample: N=84; intervention group: n=44; control group: n=40  Setting(s): Community; Primary care practices (n=13)  Country: USA  Relevance: Moderate  Quality: High | Competent Adulthood Transition with Cognitive-behavioural and Interpersonal Training programme (CATCH-IT); internet-based; 14 modules  Delivered by:  Primary care physicians trained in one hour program  Delivered to:  Youth (14 to 21 years) | To activate youth social networks and strengthen relationship skills  Closeness to parents measured with the Perceived Social Support from Family measure (Psocial support-Fa)  Social acceptance and closeness to classmates measured with Perceived Social Support from Peers (Psocial support-Fr) | Affect regulation measured with sub scales of the Center for Epidemiologic Studies Depression (CES-D)  Symptoms of other mental disorders and general health; self-rated health  Cognition and self-efficacy measured with Generalized Self-efficacy scale, self-rated intelligence, etc. |
| Vella et al. (2018)  Type: Evaluation (protocol; cluster matched control)  Sample: N=231 in each group  Setting(s): Community; sport clubs; region of Eastern Australia  Country: Australia  Relevance: Low  Quality: Moderate | Ahead of the Game; four different sports and mental promotion/ literacy programmes; one-off events e.g. 45mins workshop; group (face-to-face); internet supported modules  Delivered by:  Volunteers (who will be accredited in Mental Health First Aid); trained presenters educated in psychology; registered sport psychologist  Delivered to:  Male youth (athletes), their parents and coaches | To promote social support youth receive from parents  Youth perceived parental support subscale of the Multidimensional Scale of Perceived Social Support | Psychological distress measured with Kessler-6  Wellbeing measured with Keyes Mental Health Continuum (MHC)  Adolescent resilience measured with Connor-Davison Resilience Scale (CD-RISC) |
