# Supplementary material for: Mobilising social support to improve mental health for children and adolescents: A systematic review using principles of realist synthesis
Source: PLoS One. 2021 May 20;16(5):e0251750. doi: 10.1371/journal.pone.0251750 (PMC8136658; doi:10.1371/journal.pone.0251750)
Supplement: S1 Box — (DOCX) [file pone.0251750.s007.docx]

Box S1: Example of search strategy in PubMed

| (((child[Title] OR adolescen*[Title] OR young [Title] OR infant[Title] OR youth[Title] OR parent[Title] OR famil*[Title] OR mother[Title] OR father[Title]) AND (resil*[Title/Abstract] OR social capital[Title/Abstract] OR social support[Title/Abstract] OR social network[Title/Abstract] OR social environment[Title/Abstract] OR natural support[Title/Abstract] OR informal support[Title/Abstract]) AND (child[Title/Abstract] OR young person[Title/Abstract] OR adolescent[Title/Abstract] OR infant [Title/Abstract]) AND (interv* [Title/Abstract] OR program*[Title/Abstract] OR approach[Title/Abstract] OR support [Title/Abstract]) NOT tornado[Title/Abstract] NOT hurricane[Title/Abstract] NOT disast*[Title/Abstract] NOT HIV[Title/Abstract] NOT AIDS[Title/Abstract] NOT homeless*[Title/Abstract] NOT tuberculosis[Title/Abstract] NOT flood*[Title/Abstract] NOT bombing[Title/Abstract] NOT trauma[Title/Abstract] Sort by: Author Filters: published in the last 10 years |
| --- |
