# Supplementary material for: Mobilising social support to improve mental health for children and adolescents: A systematic review using principles of realist synthesis
Source: PLoS One. 2021 May 20;16(5):e0251750. doi: 10.1371/journal.pone.0251750 (PMC8136658; doi:10.1371/journal.pone.0251750)
Supplement: S1 File — (DOCX) [file pone.0251750.s008.docx]

Box S1: Example of search strategy in PubMed

Table S1: Assessment of relevance for included studies

Table S2: Assessment of quality of included studies

Table S3: Characteristics of included studies concerned with infants (0 to 2 years)

Table S4: Characteristics of included studies concerned with children (3 to 9 years)

Table S5: Characteristics of included studies concerned with adolescents (10 to 18 years)

Box S1: Example of search strategy in PubMed

| (((child[Title] OR adolescen*[Title] OR young [Title] OR infant[Title] OR youth[Title] OR parent[Title] OR famil*[Title] OR mother[Title] OR father[Title]) AND (resil*[Title/Abstract] OR social capital[Title/Abstract] OR social support[Title/Abstract] OR social network[Title/Abstract] OR social environment[Title/Abstract] OR natural support[Title/Abstract] OR informal support[Title/Abstract]) AND (child[Title/Abstract] OR young person[Title/Abstract] OR adolescent[Title/Abstract] OR infant [Title/Abstract]) AND (interv* [Title/Abstract] OR program*[Title/Abstract] OR approach[Title/Abstract] OR support [Title/Abstract]) NOT tornado[Title/Abstract] NOT hurricane[Title/Abstract] NOT disast*[Title/Abstract] NOT HIV[Title/Abstract] NOT AIDS[Title/Abstract] NOT homeless*[Title/Abstract] NOT tuberculosis[Title/Abstract] NOT flood*[Title/Abstract] NOT bombing[Title/Abstract] NOT trauma[Title/Abstract] Sort by: Author Filters: published in the last 10 years |
| --- |

Table S1: Assessment of relevance for included studies

| Study ID | Assessment | Rating |
| --- | --- | --- |
| Asghar et al (2018) | Increasing social support is a programme goal but social support is not defined or conceptualised; no programme theory reported; standardised measures for child wellbeing (self-esteem, confidence) but not for social support | Low |
| Ayton and Joss (2016) | Social support is specific programme goal but not defined or conceptualised; only limited theory about how to increase social support and link between social support and child and health wellbeing | Low |
| Bohleber et al (2016) | Increasing social support is specific programme goal; social support and child outcomes measured with standardised scales; consistent between hypotheses and tested relationships on social support and child outcomes but not much detail on program theory and mechanisms | Moderate |
| Byrne et al (2012) | Social support not primary programme goal; strong social support conceptualisation; comprehensive social support measure; child outcomes only measured through perceptions of child development and child rearing (no standardised measure) | Moderate |
| Branch et al (2013) | Increasing social support is primary programme goal; social support not well conceptualised and only refers to formal support provided by schools; mechanisms and theories do not directly refer to social support; child outcomes primarily refer to school related outcomes | Low |
| Cho et al (2013) | Study hypothesises important role of social support (and measures this); however, social support not part of programme theory; whilst social support measure included informal and formal support, the only social support source targeted by the intervention is support from healthcare professionals | Low |
| Cluver et al (2017) | Theory of change (depicted) includes social support but no further conceptualisation of social support; no clear programme theory; no information about expected mechanisms by which intervention would increase social support and in turn improve child outcomes; social support and child outcomes measured with standardised scales (only child behaviour and no other child mental health outcomes measured) | Low |
| Deutsch et al (2017) | Social support (conceptualised in form of mentor and peer support and relationships with friends and families) is primary goal of the programme; mechanism of how the intervention might increase social support and their refinement is subject to this study; not much detail about how social support is expected to improve child outcomes | Moderate |
| DeWit et al (2016) | Increasing social support is a programme goal but social support not defined or conceptualised; programme theory aims match well with hypothesised mechanisms, which are based on theory and evidence and well described; and also addressed in findings and conclusions; standard measures for social support and child outcomes | Moderate |
| Doty et al (2017) | Strong in conceptualisation of social support, including hypothesised pathways to social support and pathways for relationship between social support and child outcomes; however, fairly abstract with no clear explanation how this relates to a program logic or theory | Moderate |
| Drummond et al (2014) | Social support mainly refers to linkages to formal system, which is the main outcomes measure; link to informal support is made (e.g. linkages to services and school expected to increase social support for family and child) but not explained with much detail | Moderate |
| Eddy et al (2017) | Whilst authors explain social support as important intervention goal and a key mechanism for improved child mental health and development outcomes, it is not measured through standardised perceived social support scale; authors refer to this in the discussion section as possible limitation; child outcome measure via standardised mental health scales | Moderate |
| Hauken et al (2015) | Increasing social support is the primary goal of the study; social support is well conceptualised and measured using standardised scale; clear hypotheses in relation to social support and relationship with child outcomes, which are based on theory and evidence; child outcome measured with standardised scales | High |
| Ingram et al (2015) | Social support is a primary programme goal and conceptualised well (although without a definition); mechanisms provided as to how social support is changed through the intervention and how it is expected to lead to improved child outcomes | Moderate |
| January et al (2016) | Social support is a specific program aim; choice of social support measure closely aligned with programme aims; relationship to child outcomes less well explained; child / youth outcomes not measured as part of this study | Moderate |
| Lachman et al (2017) | Social support not conceptualised or defined; mechanisms shown in theory of change graph with only very short explanation to it; child outcome measured with standard scales but only one dimension (behaviour) | Low |
| Letourneau et al (2011) | Social support as specific programme aim but limited theory of how to increase social support | Moderate |
| Leventhal et al (2015) | Social support not a primary programme goal and not conceptualised; mechanisms as to how to increase social support and how this leads to improved child outcomes described only briefly; standardised child outcome measures | Low |
| Marcynyszyn et al (2011) | Social support not a primary programme goal but considered an important mediator that is being investigated in this study as a process indicator; very little explanation on how social support is expected to increase and how this would improve child outcomes; both social support and child outcomes measured with standardised tools | Low |
| Mitchell et al (2015) | Social support not explicitly conceptualised; however, it is a primary goal of the programme; mechanisms how to increase social support and how this can lead to improved child development outcomes well conceptualised | Moderate |
| Nabuco et al (2014) | Overall social support is not well conceptualised or defined; social support is not a primary programme goal; mechanisms for how the intervention increases social support and how this leads to improved child development outcomes are explained; standardised social support and child outcome measures | Moderate |
| Pancer et al (2013) | Limited hypothesis and findings in regards to social support; social support and child outcomes measured with standardised scales | Low |
| Parcel and Pennell (2012) | Increasing social support (conceptualised as family and school social capital) is primary programme goal; social support limited to family and school social capital; child outcomes relate to social adjustment, school achievements and mental health (but latter not clear how it should be measured); mechanisms to increase social support and how this leads to improved child outcomes described but without much detail | Moderate |
| Romjinders et al (2017) | Range of evidence of why social support is low in this population; focus on one mechanisms which is acceptance and tolerance but not clear how social support expected to change outside group; no clear programme theory | Moderate |
| Schwartz et al (2013) | Study provides new findings on how social support might lead to improved child outcomes; not clear to what extent this is based on expected mechanisms; most mechanisms refer to how/ why natural mentoring might increase social support but less clear how this was expected to change youth outcomes (i.e. lacks evidence on what are important mechanisms or factors for improving child or youth wellbeing) | Moderate |
| Stubbs and Achat 2016 | Increasing social support was primary programme goal and some explanation and evidence as to how this might be achieved and how this might improve child outcomes; no standardised measure for social support or child outcomes; child wellbeing only captured in terms of process indicators | Moderate |
| Swenson et al (2010) | Social support is an important part of the model, and was shown to improve post-intervention compared to controls, but it did not appear to be a significant part of the intervention as implemented, although this may be also due to reporting issues | Moderate |
| Valdez et al. (2011) | Social support not conceptualised but measured with standardised scale; programme theory does not explain how the intervention is expected to increase social support, and how social support is expected to lead to improved child outcomes; child outcomes measured with standardised scales | Low |
| Valdez et al. (2013) | Social support not conceptualised but measured with standardised scale; programme theory does not explain how the intervention is expected to increase social support, and how social support is expected to lead to improved child outcomes; child outcomes measured with standardised scales | Low |
| Van Dam et al. (2017) | Social support is conceptualised; increasing social support to achieve child outcomes is programme goal and but not much explanation as to how this is achieved (assumption that providing a mentor will lead to this increased support); not much detail on mechanisms to improved child outcomes (and which ones specifically) social support and child outcomes not measured with standardised scales | Moderate |
| Van Voorhees et al. (2008) | Social support is one of several programme goals but has not been conceptualised; some, but not much, information about how to improve social support and how social support improves child outcomes; social support and child outcomes measured with standardised scales | Moderate |
| Vazquez et al. (2017) | Social support measured with a standardised tool but not conceptualised and unclear whether it is considered a primary programme goal; there is also no programme theory as to how social support is expected to improve child outcomes; in terms of child outcomes only child behaviour measured with standardised sub scale; | Low |
| Vella et al. (2018) | Social support increase is underlying programme goal but not conceptualised; mechanisms for increasing social support and achieving child outcomes not well described; Social support and child outcomes measured with standardised scales | Low |

Table S2: Assessment of quality of included studies

| Quantitative randomised controlled trials | | | | | | | | | | | |
| --- | --- | --- | --- | --- | --- | --- | --- | --- | --- | --- | --- |
| Study ID | Randomisation appropriately performed | | Groups comparable at baseline | | Complete outcomes data | | Outcome assessor blinded | | Adherence to intervention | | Summary rating |
| Drummond et al (2014) | Yes | | Not yet known | | Not yet known | | Yes | | Not yet known | | High (expected based on study protocol) |
| Eddy et al (2017) | No | | Can’t tell | | No | | No | | No | | Low |
| Hauken et al (2015) | No | | Not yet known | | Not yet known | | No | | Not yet known | | Moderate (expected based on study protocol) |
| Lachman et al (2017) | Yes | | Yes | | Yes | | Yes | | Yes | | High |
| Letourneau et al (2011) | No | | Yes | | No | | Yes | | No | | Moderate |
| Leventhal et al (2015) | Yes | | Yes | | Yes | | No | | Yes | | High |
| Swenson et al (2010) | Yes | | Yes | | Yes | | No | | Yes | | High |
| Van Voorhees et al (2008) | Yes | | Yes | | Yes | | No | | Yes | | High |
| Quantitative non-randomised studies | | | | | | | | | | | |
|  | Participants representative of target population | | Measurements appropriate regarding both the outcome and the intervention/ exposure | | Outcomes data complete | | Confounders accounted for in analysis | | Intervention administered as intended | | Summary rating |
| Asghar et al (2018) | Can’t tell | | No | | No | | No | | No | | Low |
| Bohleber et al (2016) | No | | Yes | | No | | Yes | | No | | Low |
| Byrne et al (2012) | No | | Yes | | Can’t tell | | Yes | | Yes | | Moderate |
| Cho et al (2013) | No | | Yes | | No | | No | | Yes | | Moderate |
| Cluver et al (2017) | Yes | | No | | Yes | | No | | Yes | | Moderate |
| DeWit et al (2016) | Yes | | Yes | | No | | Yes | | Can’t tell | | Moderate |
| January et al (2016) | Can’t tell | | No | | Can’t tell | | Yes | | Yes | | Low |
| Marcynyszyn et al (2011) | Yes | | Yes | | Yes | | No | | No | | Moderate |
| Nabuco et al (2014) | Can’t tell | | Can’t tell | | Can’t tell | | Yes | | Yes | | Moderate |
| Pancer et al (2013) | Can’t tell | | Yes | | Yes | | Yes | | Yes | | High |
| Schwartz et al (2013) | Yes | | No | | Yes | | Yes | | Can’t tell | | Moderate |
| Stubbs and Achat (2016) | No | | No | | Can’t tell | | No | | No | | Low |
| Valdez et al (2011) | Can’t tell | | No | | No | | No | | No | | Low |
| Valdez et al (2013) | Can’t tell | | Yes | | No | | No | | Yes | | Moderate |
| Van Dam et al (2017) | Yes | | No | | Yes | | No | | No | | Moderate |
| Vazquez et al (2017) | Can’t tell | | Can’t tell | | No | | No | | Can’t tell | | Low |
| Vella et al (2018) | Can’t tell | | Yes | | Not yet known | | Yes | | Not yet known | | Moderate (expected based on study protocol) |
| Qualitative studies | | | | | | | | | | | |
| Study ID | Appropriate qualitative approach | | Adequate data collection methods | | Findings adequately derived from data | | Interpretation of results sufficiently substantiated by data | | Coherence between data sources, collection, analysis and interpretation | | Summary rating |
|  |  | |  | |  | |  | |  | |  |
| Asghar et al (2018) | Yes | | Yes | | Yes | | No | | No | | Moderate |
| Ayton and Joss (2014) | Yes | | No | | Yes | | Yes | | Yes | | High |
| Branch et al (2013) | Yes | | No | | Can’t tell | | No | | No | | Low |
| Deutsch et al (2017) | Yes | | Yes | | Yes | | Yes | | Yes | | High |
| Mitchell et al (2015) | Can’t tell | | No | | Can’t tell | | Can’t tell | | Can’t tell | | Low |
| Romjinders et al (2017) | Yes | | Yes | | Yes | | Yes | | Yes | | High |
| Schwartz et al 2013 | Yes | | Yes | | Yes | | Yes | | Yes | | High |
| Vazquez et al (2017) | Yes | | Can’t tell | | Can’t tell | | Can’t tell | | Can’t tell | | Low |
| Mixed methods studies | | | | | | | | | | | |
|  | Adequate rationale for using mixed method | Different components effectively integrated | | Outputs of the integration of qual. and quant. components adequately interpreted | | Divergences and inconsistencies between quant. And qual. results adequately addressed | | Different components adhere to quality criteria of each tradition of methods involved | | Summary score | |
| Asghar et al (2018) | No | Yes | | No | | Yes | | No | | Moderate | |
| Schwartz et al (2013) | Yes | Yes | | Yes | | Yes | | No | | High | |

Table S3: Characteristics of included studies concerned with infants (0 to 2 years)

| Study details | Intervention characteristics | Social support aim(s) and measure(s) | Child outcome measure(s) |
| --- | --- | --- | --- |
| Cho et al (2013)  Type: Evaluation (non-randomised control group design)  Size: N=66 (intervention group: n=26; control group: n=40)  Setting(s): Hospital; 3 hospitals with Growing Care Units, Tokyo  Country: Japan  Relevance: Low  Quality: Moderate | Hospital and home visiting program ‘The Japanese Infant Mental Health Program’ (JIMHP); JIMHP helpers meet with mother once in hospital and 5 times at home  Delivered by:  JIMH helpers (clinicians) trained by European Early Promotion Project; 10 workshops (including role play e.g. active listening)  Delivered to:  Mothers of infants (pre-term - until 12 months of corrected age) | To promote mothers’ social support (mainly informational support from relationships with healthcare professionals)  Support from partner, family, friends, health professionals measured with modified version of Social Support Scale | Postural-motor, cognitive-adaptive, and language-social skills measured with standardized child development test |
| Letourneau et al (2011)  Type: Evaluation (RCT)  Size: N=60 (intervention group: n=27; control group: n=33)  Setting(s): Community; recruited via campaign and health professionals in two Canadian provinces (Alberta and New Brunswick)  Country: Canada  Relevance: Moderate  Quality: Moderate | Home-based peer support; included maternal–infant interaction teaching (Keys to Caregiving programme); 12 weeks  Delivered by:  Volunteers (mothers recovered from postpartum depression); 8-hour classroom-based sessions and regular follow-up, debriefing  Delivered to:  Mothers with depression of infants (<9 months) | To increase maternal social support, including her perceptions thereof  Perceived support (guidance, reliable alliance, reassurance of worth, attachment, social integration, opportunity for nurturance) measured with Social Provisions Scale | Maternal reports of social–emotional development measured with Infant Characteristics Questionnaire  Stress measured with cortisol levels |
| Mitchell et al (2015)  Type: Evaluation (qualitative)  Size: N=20  Setting(s): Public hospital specialist maternity service; north east region of Melbourne  Country: Australia  Relevance: Moderate  Quality: Low | Community-based pilot program of volunteer home visiting (‘Mentoring Mums’); volunteers walks alongside isolated mother from late pregnancy/ early infancy for period of up to 2 years  Delivered by:  Community volunteers (mothers); 3-days training program and ongoing sessions; support from coordinator and caseworker, who also works with mother  Delivered to:  Mothers of infants | To help mothers engage with local services and supports by modelling friendliness and providing opportunities for practicing social skills  Changes in social isolation measured in form of changes in access to services and interaction with formal (health) service systems, and the local community (various data sources) | Child development across a range of milestones; increased mother-infant attachment (various data sources) |
| Stubbs and Achat (2016)  Type: Evaluation (pre post design)  Size: N=118 (baseline), N=65 (follow up)  Setting(s): Community health centre in western Sydney, North South Wales (NSW)  Country: Australia  Relevance: Moderate  Quality: Moderate | ‘Family Partnership Model’; delivered through home visits; average 2.2 direct contacts per month (median 60 minutes); duration min. 6 months  Delivered by:  Child and family health nurses trained in Family Partnership Model  Delivered to:  Parents of infants (median age 7 weeks) | To link isolated families to services and enhancing community and support networks, and offer social support  Parents’ perceived support from interpersonal relationships; given + received formal and informal support (various questions + data sources) | Questions whether/ how child development was impacted by program |

Table S4: Characteristics of included studies concerned with children (3 to 9 years)

| Study details | Intervention characteristics | Social support measure | Child outcome measure(s) |
| --- | --- | --- | --- |
| Ayton & Joss (2016)  Type: Evaluation (qualitative)  Size: Mentors n=27; Mentees n=12  Setting(s): Community; two church sites, Mornington Peninsula, Victoria  Country: Australia  Relevance: Low  Quality: High | Home-visiting mentoring program (‘Creating Opportunities and Casting Hope’; COACH) for vulnerable parents; duration 12 months  Delivered by:  Volunteers (church attendees) interviewed and trained by program staff who are social workers (14 hours); plus mentor training manual  Delivered to:  Parents of children < 12 years | To help parent to reach own goals related to social support such as developing relationships with others and establishing community connections  Parent’s perceived benefits of mentoring support received; goals achieved in regards to social support goal | Questions about health and wellbeing of children |
| Branch et al (2013)  Type: Evaluation (qualitative)  Sample: Semi-structured interviews (n=22) with parents, teachers, program staff; survey (n=20) with parents and teachers  Setting(s): Residential sites (no details provided)  Country: Australia  Relevance: Low  Quality: Low | Circles of Care; involves regular meetings where all members of the Circle participate (ideally once per school term)  Delivered by:  Community workers, which are trained, coached and supervised by a coordinator  Delivered to:  Children (5 to 11 years), children’s family, teachers, community workers, others | To enhance support networks around child and provide access to formal social support, including through school integration  Questions about parents’ support from school, community welfare services, and their participation; support and assistance received by child | Questions about improvements in child behaviour |
| Byrne et al 2012  Type: Evaluation (pre post design)  Sample: N=494  Setting(s): Community; family program in regions of Castile and Leon  Country: Spain  Relevance: Moderate  Quality: Moderate | Parenting programme ‘The Personal Family and Family Support program’ Apoyo Personal y Familiar (APF); 90 min session per week for 8 months; plus two warm up sessions at start of program; group based  Delivered by:  Facilitators (locally trained social workers; trained 25 hours before program; additional training/ supervision session half way through program)  Delivered to:  Parents of children (age not specified) | To reinforce parents’ perception of and increase satisfaction with social support networks  Various sources of informal and formal social support measured with modified Scale of Social Support in Informal and Formal systems and Social Support Questionnaire | Parents’ beliefs around child development measured with questionnaire  Various aspects of child rearing, parental agency (measured with standardised scales) |
| Doty et al (2017)  Study type: Conceptual (programme theory development)  Sample: Not applicable  Setting(s): Not applicable  Country: USA  Relevance: Moderate  Quality: Not applicable | Holistic parenting interventions focused on strengthening parent-child relationship and building family social capital  Delivered by:  Practitioners (not specified)  Delivered to:  Parents of children (age not specified) | To help parents build social networks by increasing their confidence to mobilise support and build social capital for child  Social support conceptualised as long-term social resources available to family | Expected improvements in child development, wellbeing and resilience |
| Drummond et al. (2014)  Type: Evaluation (protocol; RCT)  Sample: N=1,173 (n=291 to 293 in each of three intervention groups; control group: n=291)  Setting(s): Community; City of Edmonton; recruitment via campaign and welfare programs  Country: Canada  Relevance: Moderate  Quality: High | Families First Edmonton (FFE), service integration approaches for low income families; 1.4 to 4.6 hours per month; duration 18 to 24 months  FFE described as complex programs provided from multi-level and sectoral collaborations; incorporate principles of family-centredness, cultural sensitivity, capacity building and reflection  Delivered by:  Families Matter partnership of four pre-existing community services  Delivered to:  Families with at least one child <12 years (study protocol , no mean age) | To increase formal social support of families, including through service integration (e.g. school and childcare)  Family linkage to services and resources measured with Family Services Inventory (FSI)  Social functions and provisions from relationships with others measured with Social Provision Scale (SPS) | Child psychosocial health measured with Behavioral Assessment System for Children (BASC) |
| Eddy et al (2017)  Type: Evaluation (multi-site RCT; growth curve analysis over 5 years)  Sample: N=278 children, and one of their caregivers; Year 5: N=184 (intervention group: N=109; control group: N=75)  Setting(s): Public elementary schools in Boston, New York City, Portland (Oregon), Seattle  Country: USA  Relevance: Moderate  Quality: Low | Paid professional mentoring program ‘Friends of the Children’ (FOTC); average 4 hours of mentoring per week (plus additional activities); average duration 4.4 years; one-to-one and group activities  Delivered by:  Mentors (organised through non-profit community-based organisations); 1-week training and ongoing supervision by FOTC mentors as well as FOTC programme director and external consultants  Delivered to:  Children (mean age 6.5 years) | To provide a child with social support opportunities; including access to material and practical support (e.g. health care, schooling)  Mentors asked about amount of contact and specific activities they had with or for child | Scales from Child Behaviour Checklist (CBCL)  Behaviour and Emotional Rating Scale (BERS-2)  Child reported antisocial behaviour scale and deviant peers scale |
| Ingram et al (2015)  Type: Conceptual (description of programme theory)  Sample: Not applicable  Setting: Boys Town (child welfare treatment provider), District of Columbia  Country: USA  Relevance: Moderate  Quality: Not applicable | Type of intervention:  In-home family service model; family goals established and plans put in place to meet those; Social Network Map used to formalise initial assessment and assist with service planning; on average n=105 days per family  Delivered by:  Caseworkers with access to web-based integrated assessment and service system  Delivered to:  Parents of children (age not specified) | To increase informal and formal supports for families that help them maintain progress after formal services ended; by teaching them skills to ask for and utilise social support  Various formal and informal social support perceived and received by family measured with Social Network Map | Child behaviour, wellbeing  Perceived levels of stress related to social support system (e.g. Strengths and Stressors tool) |
| Lachman et al. (2017)  Type: RCT  Sample: N=68 (intervention group: n=34; control group: n=34)  Setting(s): Community; Khayelitsha (suburban region of Cape town)  Country: South Africa  Relevance: Low  Quality: High | Parent training program (Sinovuyo Caring Families Program); 12 weekly session; each 2-3 hours; 10-14 parents per group; home consultations when parents miss session  Delivered by:  Community-based facilitators trained by program staff  Delivered to:  Parents of children aged 3 to 8 years | To improve parent’s social support  Parent’s perceived social support (various sources) measured with Multidimensional Scale of Perceived Social Support (MSPsocial support) | Child behaviour measured through Sinovuyo Observational  Coding System (SOCS) and Eyberg Child Behavior Inventory (ECBI) |
| Marcynyszyn et al (2011)  Type: Evaluation (pre post)  Sample: N=41  Setting(s): Two child welfare agencies (Casey Family Program), New York State  Country: USA  Relevance: Low  Quality: Moderate | Group-based parent training and education program ‘Incredible Years’ (IY); 12 to 14 weeks duration; 2 hours each week  Delivered by:  Group leaders who are therapists; trained in 2 day workshop by IY program developers; clinical supervision  Delivered to:  Parents of children aged between 6 months and 8 years | To improve perceived social support of parents  Parents’ perceived social support (various sources) measured with Multidimensional Scale of Perceived Social Support (MSPsocial support) and Family Support Scale (Fsocial support) | Child behaviour measured in previous trials (improvements well established) |
| Nabuco et al (2014)  Type: Evaluation (cross sectional, quasi-experimental)  Sample: 2008: N=103 (intervention group: N=57, control group=46); 2009: N=142 (intervention group: N=71, control group: N=71); 2010: N=187 (intervention group=92, control group=95)  Setting(s): Community; A-PAR program setting and recruitment from crèches and nurseries; Greater Lisbon  Country: Portugal  Relevance: Moderate  Quality: Moderate | Early childhood education and parenting support (A-PAR); based on UK’s Parents Early Education Partnership (PEEP); one-hour weekly group sessions with parents (or significant other) and child; provided for one year  Delivered by:  Leaders with four year university degree in early childhood education; trained 25 hours by program developer; ongoing training (2 hours per month) supervision by program staff  Delivered to:  Parents of children (up to 6 years) | To convince parents of benefit of social support for child welfare and to promote their social support networks  Parents’ social support measured with Social Support Questionnaire (QAS) | Socio-emotional development measured with Social Competence Scale (QCS) and Emotional Activity and Sociability Scale (EEAS)  Cognitive development measured with Wechsler Preschool and Primary Scale of Intelligence-Revised (WPPSI) |
| Pancer et al (2013)  Type: Evaluation (cohort design; follow up when youth were 18 to 19 years)  Sample: N=626 (intervention group: N=401; control group: N=225)  Setting(s): Community; schools in three regions; Ontario  Country: Canada  Relevance: Low  Quality: High | Better Beginnings, Better Future (BBBF); community-driven programmes offering wide range of education, play, sports, arts or leisure activities  Delivered by:  Various members of the community, parents, children  Delivered to:  Parents and their children (4 to 8 years) | To help parents gain access to social and tangible support  Parents’ social support measured with Social Provisions Scale  Other aspects of parents’ perceived social support measured with single questions | Children’s social behaviours measured on subscales from the Social Skills Rating Scale  Cognitive and academic performance (various measures) |
| Parcel and Pennell (2012)  Type: Conceptual (programme theory development)  Sample: Not applicable  Setting(s): Community; schools  Country: USA  Relevance: Moderate  Quality: Not applicable | Child and family teams (CFTs) - teams around child to plan how to support child and family  Delivered by:  CFT facilitators and other involved school staff  Delivered to:  Children (school age), (extended) family, school teachers | To wrap a comprehensive and unified array of services and supports around children and their families | Child development, mental health and wellbeing  Social adjustment and behaviour; crimes and acting out |
| Vazquez et al. (2017)  Type: Evaluation (pre post)  Sample: N=216 (baseline); N=130 (follow up)  Setting(s): Community; various recruitment strategies; Barcelona  Country: Spain  Relevance: Low  Quality: Low | Parenting Skills Program for families (PSP); 11 weekly sessions of 90 minutes; groups of 10-14 parents (children participate in two sessions)  Delivered by:  Practitioners in community services; trained in PSP  Delivered to:  Parents with children (aged 2 to 12 years; most children between 3 and 5) | To increase parents’ perceived social support  Parents’ perceived social support (confident and emotional) measured with Duke-UNC Functional Social Support Questionnaire  Open questions about social support to parent | Child behaviour through sub-scale of Strength and Difficulties Questionnaire  Children’s emotional self-regulation through single questions  Children behaviour effects through open questions |

Table S5: Characteristics of included studies concerned with adolescents (10 to 18 years)

| Study details | Intervention characteristics | Social support aim(s) and measure(s) | Child outcome measure(s) |
| --- | --- | --- | --- |
| Asghar et al. (2018)  Type: Evaluation (mixed method)  Sample: Quant.: N=78; Qual.: N=15  Setting(s): Community and camps; Women Community Centres (n=9); four located in camps for displaced people; different districts of Khyber-Pakhtunkwa region  Country: Pakistan  Relevance: Low  Quality: Moderate | Creating Opportunities through Mentorship, Parental Involvement, and Safe Spaces (COMPAsocial support) program; weekly 45 to 60 minutes group sessions plus 30 mins unstructured time; 26 sessions for girls; 14 sessions for female caregivers  Delivered by:  Mentors employed by implementing organisations, which received targeted training and ongoing support from program developers  Delivered to:  Adolescent girls (12 to 19 years) and their female caregivers | To increase girls’ social assets over time (including friends and trusted adults)  Quality of relationship with caregivers measured in comfort talking about intimate topics: education, earning a living, marriage, puberty | Self-esteem measured with Rosenberg Self-esteem Scale  Hope measured with Children's Hope Scale  Self-reported school attendance |
| Bohleber et al. (2016)  Type: Evaluation (non-randomised control group design)  Sample: 1^st^ sample youth in employment: intervention group: N=546; control group: N=395; 2^nd^ sample of youth in unemployment: intervention group: N=73; control group: N=120  Setting(s): Swiss company and public employment transition program  Country: Switzerland | Mental health promotion app (‘Companion App’), which provides peer mentoring system, group discussions, links to websites on mental health issues and leisure activities information, anonymous professional counselling service; provided over 10 months  Development in consultation with youth focus group and users have opportunity to provide feedback and suggest improvements  Delivered by:  Peer mentors (no further details provided on how they were trained); research team developed and maintained the App  Delivered to:  Young people (mean age 17 years) | To increase adolescents’ perceived social support; to enhance their feelings of connectedness to school and parents  Satisfaction with social support and reciprocity in social support measured with two scales of Social Support Questionnaire | *Chronic stress measured with Trier Inventory of Chronic Stress screening scale (TICS-SCsocial support)* |
| Cluver et al (2017)  Type: Evaluation (pre post; pilot)  Sample: N=60  Setting(s): Community; community-based organisation in rural area of Eastern Cape province  Country: South Africa  Relevance: Low  Quality: Moderate | Parenting program ‘Sinovuyo Caring Families Teen Programme’ (Sinovuyo = we have happiness or joy); 10-sessions  Delivered by:  Community staff with experience of conducting parenting programmes; trained 1 week on collaborative learning techniques, modelling praise and problem solving skills  Delivered to:  Children (aged 10 to 17 years) and their mothers or female caregivers | To improve parent and adolescent social support  Parents’ and youth’s social support (emotional, tangible, affectionate) and positive social interaction measured with the Medical Outcome Study Social Support Survey | Adolescent behaviour problems measured using subscales of the Child Behaviour Checklist (CBC) |
| Deutsch et al (2017)  Type: Evaluation (qualitative)  Sample: N=113  Setting(s): Community; schools  Country: USA  Relevance: Moderate  Quality: High | Youth mentoring programme (Young Women Leaders Program, YWLP); one-to-one and group component; frequency or duration not specified  Delivered by:  Women mentors (volunteers); training (amount, duration not specified)  Delivered to:  Children (mean age 12) | To optimise social support  Qualitative exploration of expanded peer networks through making new friends as well as becoming closer to people they already knew | Youth development (social, emotional, cognitive, identity)  Qualitative exploration of self-regulation |
| DeWit et al (2016)  Type: Evaluation (cohort; 18 months follow up)  Sample: N=859  Setting(s): Community; 21 Big Brothers Big Sisters agencies across Canada  Country: Canada  Relevance: Moderate  Quality: Moderate | Youth mentoring ‘Big Brothers Big Sisters’ community mentoring relationship’ (BBBS); 2 to 4 hours per week for period of one year  Delivered by:  Mentors (volunteers) and caseworkers trained by programme staff  Delivered to:  Adolescents (up to 17 years) | To strengthen, nurture and support relationships of young persons with caring and responsible adults  Perceived peer and teacher support via subscales of the Social Support Appraisal Scale (SSAS)  Perceived parent emotional support measured with sub scale of the Wills Parental Support Scale (WPSS) | Various mental health problems measured wit Strengths and Difficulties Questionnaire (SDQ)  Depression, anxiety, stress measured with: Generalized Social Anxiety and Distress sub-scale (SAD-G) of Revised Social Anxiety Scale for Children (SASC-R), subscale from Center for Epidemiology Studies Depression Scale (CES-DC) |
| Hauken et al. (2015)  Type: Evaluation (protocol; RCT)  Sample: Planned: N=120 parents (n=60); children (n=60); equal allocation between IG and CG  Setting(s): Community; national recruitment strategy including cancer charities, wider range of health professionals  Country: Norway  Relevance: High  Quality: Moderate | Psycho-educational program for the social network (‘PEPSONE’) approx. 3 hours session at families’ home  Delivered by:  Clinical psychologists  Delivered to:  Family with child aged 8-18 years; social network members (study protocol, no mean age) | To optimize social network support of families whose children live with cancer  Parents’ social support after crisis measured with the Crisis Support Scale (CSS)  Parents and social network members received/ provided social support measured with the Assistance Questionnaire-Receivers / Providers of support (AQR/ AQP) | Anxiety measured with Revised Child Manifest Anxiety Scale (RCMAS)  Quality of life measured with the Kinder Lebensqualität (KINDL) |
| January et al (2016)  Type: Evaluation (pre post design)  Sample: N=139  Setting(s): Community organisation in Western United States  Country: USA  Relevance: Moderate  Quality: Low | Peer-to-peer support prevention program delivered via telephone (Parent Connectors); Family contact log completed, reviewed and updated by Parent Connector; duration: 3 months  Delivered by:  Volunteers; trained 3 days through a manualized training curriculum and weekly 2 hours supervision sessions by trained mental health practitioner  Delivered to:  Parents of children (mean age 11 years) | To promote positive attitudes toward building social support networks; to provide emotional support to reduce feelings of blame and stigma; instrumental support to reduce basic needs such as clothing, food; informational support to increase academic and behavioral success  Perceived informal support from others and perceived access to services and tangible goods to assist the family in coping with stress measured with social and concrete support domains of the Protective Factors Survey (PFS) | Children outcomes not reported in this study but previous evaluation of the same intervention provided to youth measured mental health with the Strengths and Difficulties Questionnaire (SDQ) and Brief Impairment Scale (BIS) |
| Leventhal et al. (2015)  Type: RCT (evaluation)  Sample: At follow-up N=2,387 (intervention group: n=1,681; control group: n=706)  Setting(s): Community; Government schools (n=57), Bihar  Country: India  Relevance: Low  Quality: High | Resilience-framework-based intervention (Girls First Resilience Curriculum; RC); over the course of 5 months; 23 weekly facilitated peer-support sessions over 5 months; group based (12-15 girls)  Delivered by:  Local women with at least 10^th^ grade education as group facilitators; 5 days initial and 3 days follow up training by Master Trainers; supervision and refresher training  Delivered to:  Girls (mean age 13 years) | To change perception of benefit of helping each other, social skills to do so and to increase links with peers and community  Extent to which child/ adolescent feels supported and accepted by peers and friends measured with the Social Support and Peers subscales of Kidscreen-52 | Depression with Patient Health Questionnaire-9 (PHQ-9)  Anxiety with General Anxiety Disorder-7 (GAD-7)  Positive psychological sub scale of KIDSCREEN-52  Social-emotional assets with items from the Child and Youth Resilience Measure |
| Romjinders et al (2017)  Study type: Evaluation (qualitative)  Sample: N=12  Setting: Community; drop in program, Houston, Texas  Country: USA  Relevance: Moderate  Quality: High | Drop-in program for sexual and gender minority (SGM) youth (Hatch Youth); drop-in meetings (3 hours, 3 nights a week), youth-led peer support group & mentoring; average duration: 14.5 months  Delivered by:  Volunteers (or sometimes staff) who are trained as group facilitators; youth themselves  Delivered to:  Youth (mean age 18 years) | To increase social support from primary and secondary social ties  Explored qualitatively (emerged as dominant theme during interviews) | Youth confidence and self-esteem explored qualitatively |
| Schwartz et al (2013)  Type: Evaluation (mixed methods)  Sample: Quant. N=1,173; qual. N=30  Setting(s): National Guard Youth Challenge Program sites (n=10) across the country  Country: USA  Relevance: Moderate  Quality: High | Youth initiated mentoring (YIM); weekly contacts between mentor and mentees; at least two face-per-face meetings per month; formal participation: 1 year  Delivered by:  Volunteers (natural mentors), interviewed and trained by program staff  Delivered to:  Youth (16 to 18 years) | To optimise role of social support through caring adult  Different aspects of social support explored qualitatively  Mentoring relationships (frequency, duration and type of contact) through survey questions | Youth educational, vocational, and behavioural outcomes explored qualitatively |
| Swenson et al. (2010)  Type: Evaluation (RCT)  Sample: N=86  Setting(s): Community; Child Protection Services, Charleston County  Country: USA  Relevance: Moderate  Quality: High | ‘Multi-systemic Therapy (MST) – daily individual sessions; duration min. 6 months; 24/7 availability for crises management  Delivered by:  Therapists including MST-trained psychiatrist; MST supervisor  Delivered to:  Families with children 10 to 17 years (mean age 14 years) | To increase (and optimise) social support  Parents’ social support in categories i.e. perceived, appraisal and belonging measured with Interpersonal Support Evaluation List (ISEL) | Youth behavioral and emotional functioning with Child Behavior Checklist (CBCL)  Trauma Symptom Checklist for Children (TSCC)  Parent completed Social Skills Rating System |
| Valdez et al. (2011)  Type: Evaluation (pre post; non-experimental; pilot)  Sample: N=10 mothers; N=16 children  Setting(s): Community; two outpatient mental health clinics, Greater Baltimore, Maryland  Country: USA  Relevance: Low  Quality: Low | Keeping Families Strong (KFS) for families in which mothers has depression; 10 multi-family group sessions  Delivered by:  Adult and children’s mental health clinicians (supervised by research clinicians)  Delivered to:  Mothers in treatment for depression, their partners and children (aged between 9 and 16 years) | To increase interfamily social support  Mothers’ perceived social support (various sources) measured with Multidimensional Scale of Perceived Social Support  Quality of mothers’ intimate relationships measured with Dyadic Adjustment Scale | Behaviour Assessment System for Children (BASC)  Social emotional competence, resilience and coping measured with Child Coping Strategies Checklist (CCSC)  Coping Efficacy Scale |
| Valdez et al. (2013)  ype: Evaluation (pre post; feasibility)  Sample: N=13 families  Setting(s): Community; three community outpatient clinics located in Latino neighbourhoods; middle sized city in the Midwest  Country: USA  Relevance: Low  Quality: Low | Fortalezas Familiares (Family Strengths); adapted from Keeping Families Strong (KSF); 12-week multi-family intervention plus two booster meetings  Delivered by:  Research clinicians in a community agency  Delivered to:  Mothers in treatment for depression, other caregivers and children (aged 9 to 18 years) | To increase interfamily social support  Mothers’ perceived social support (various sources) measured with Multidimensional Scale of Perceived Social Support | Child psychological functioning measured with the Strengths and Difficulties Questionnaire (SDQ)  Child coping measured with subscales of the Children’s Coping Strategies Checklist Revision 1 (CCSC) and Coping Efficacy Scale (CES) |
| Van Dam et al. (2017)  Type: Evaluation (case analysis; cross sectional study)  Sample: N=200 (intervention group: n=96; control group: n=104)  Setting: Residential; youth organisations providing residential care  Country: Netherlands  Relevance: Moderate  Quality: Moderate | ‘Youth Initiated Mentoring’ (YIM); various meetings take place between YIM, young person, family and professionals; duration between 6 and 9 months  Delivered by:  Volunteers (natural mentors)  Delivered to:  Youth (12 to 23 years) | To increase youth collaboration with the family and its social network; to help youth request and maintain support from others  Received social support (emotional, practical, guidance and advice) measured through data records | Youth health and mental wellbeing measured with CAP-J  Dutch classification instrument for youth mental health |
| Van Voorhees et al. (2008)  Type: Evaluation (RCT)  Sample: N=84; intervention group: n=44; control group: n=40  Setting(s): Community; Primary care practices (n=13)  Country: USA  Relevance: Moderate  Quality: High | Competent Adulthood Transition with Cognitive-behavioural and Interpersonal Training programme (CATCH-IT); internet-based; 14 modules  Delivered by:  Primary care physicians trained in one hour program  Delivered to:  Youth (14 to 21 years) | To activate youth social networks and strengthen relationship skills  Closeness to parents measured with the Perceived Social Support from Family measure (Psocial support-Fa)  Social acceptance and closeness to classmates measured with Perceived Social Support from Peers (Psocial support-Fr) | Affect regulation measured with sub scales of the Center for Epidemiologic Studies Depression (CES-D)  Symptoms of other mental disorders and general health; self-rated health  Cognition and self-efficacy measured with Generalized Self-efficacy scale, self-rated intelligence, etc. |
| Vella et al. (2018)  Type: Evaluation (protocol; cluster matched control)  Sample: N=231 in each group  Setting(s): Community; sport clubs; region of Eastern Australia  Country: Australia  Relevance: Low  Quality: Moderate | Ahead of the Game; four different sports and mental promotion/ literacy programmes; one-off events e.g. 45mins workshop; group (face-to-face); internet supported modules  Delivered by:  Volunteers (who will be accredited in Mental Health First Aid); trained presenters educated in psychology; registered sport psychologist  Delivered to:  Male youth (athletes), their parents and coaches | To promote social support youth receive from parents  Youth perceived parental support subscale of the Multidimensional Scale of Perceived Social Support | Psychological distress measured with Kessler-6  Wellbeing measured with Keyes Mental Health Continuum (MHC)  Adolescent resilience measured with Connor-Davison Resilience Scale (CD-RISC) |

Asghar, K., Mayevskaya, Y., Sommer, M., Razzaque, A., Laird, B., Khan, Y., et al. (2018). Promoting Adolescent Girls' Well-Being in Pakistan: a Mixed-Methods Study of Change Over Time, Feasibility, and Acceptability, of the COMPASS Program. *Prev Sci*.

Ayton, D., & Joss, N. (2016). Empowering vulnerable parents through a family mentoring program. *Aust J Prim Health,* 22, 320-326.

Branch, S., Homel, R., & Freiberg, K. (2013). Making the developmental system work better for children: lessons learned implementing an innovative programme. *Child & Family Social Work,* 18, 294-304.

Byrne, S., Rodrigo, M.J., & Martin, J.C. (2012). Influence of form and timing of social support on parental outcomes of a child-maltreatment prevention program. *Children and Youth Services Review,* 34, 2495-2503.

Cho, Y., Hirose, T., Tomita, N., Shirakawa, S., Murase, K., Komoto, K., et al. (2013). Infant Mental Health Intervention for Preterm Infants in Japan: Promotions of Maternal Mental Health, Mother-Infant Interactions, and Social Support by Providing Continuous Home Visits until the Corrected Infant Age of 12 Months. *Infant Mental Health Journal,* 34, 47-59.

Cluver, L.D., Lachman, J.M., Ward, C.L., Gardner, F., Peterson, T., Hutchings, J.M., et al. (2017). Development of a Parenting Support Program to Prevent Abuse of Adolescents in South Africa: Findings From a Pilot Pre-Post Study. *Research on Social Work Practice,* 27, 758-766.

Deutsch, N.L., Reitz-Krueger, C.L., Henneberger, A.K., Ehrlich, V.A.F., & Lawrence, E.C. (2017). "It Gave Me Ways to Solve Problems and Ways to Talk to People": Outcomes From a Combined Group and One-on-One Mentoring Program for Early Adolescent Girls. *Journal of Adolescent Research,* 32, 291-322.

DeWit, D., DuBois, D., Erdem, G., Larose, S., Lipman, E., DeWit, D.J., et al. (2016). The Role of Program-Supported Mentoring Relationships in Promoting Youth Mental Health, Behavioral and Developmental Outcomes. *Prevention Science,* 17, 646-657.

Doty, J.L., Davis, L., & Arditti, J.A. (2017). Cascading Resilience: Leverage Points in Promoting Parent and Child Well-Being. *Journal of Family Theory & Review,* 9, 111-126.

Drummond, J., Schnirer, L., So, S., Mayan, M., Williamson, D.L., Bisanz, J., et al. (2014). The protocol for the Families First Edmonton trial (FFE): a randomized community-based trial to compare four service integration approaches for families with low-income. *BMC Health Serv Res,* 14, 223.

Eddy, J., Martinez, C., Grossman, J., Cearley, J., Herrera, D., Wheeler, A., et al. (2017). A Randomized Controlled Trial of a Long-Term Professional Mentoring Program for Children at Risk: Outcomes Across the First 5 Years. *Prevention Science,* 18, 899-910.

Hauken, M.A., Senneseth, M., Dyregrov, A., & Dyregrov, K. (2015). Optimizing Social Network Support to Families Living With Parental Cancer: Research Protocol for the Cancer-PEPSONE Study. *JMIR Res Protoc,* 4, e142.

Ingram, S.D., Cash, S.J., Oats, R.G., Simpson, A., & Thompson, R.W. (2015). Development of an evidence-informed in-home family services model for families and children at risk of abuse and neglect. *Child & Family Social Work,* 20, 139-148.

January, S.-A., Duppong Hurley, K., Stevens, A., Kutash, K., Duchnowski, A., & Pereda, N. (2016). Evaluation of a Community-Based Peer-to-Peer Support Program for Parents of At-Risk Youth with Emotional and Behavioral Difficulties. *Journal of Child & Family Studies,* 25, 836-844.

Lachman, J.M., Cluver, L., Ward, C.L., Hutchings, J., Mlotshwa, S., Wessels, I., et al. (2017). Randomized controlled trial of a parenting program to reduce the risk of child maltreatment in South Africa. *Child Abuse Negl,* 72, 338-351.

Letourneau, N., Stewart, M., Dennis, C.L., Hegadoren, K., Duffett-Leger, L., & Watson, B. (2011). Effect of home-based peer support on maternal-infant interactions among women with postpartum depression: a randomized, controlled trial. *Int J Ment Health Nurs,* 20, 345-357.

Leventhal, K.S., Gillham, J., DeMaria, L., Andrew, G., Peabody, J., & Leventhal, S. (2015). Building psychosocial assets and wellbeing among adolescent girls: A randomized controlled trial. *J Adolesc,* 45, 284-295.

Marcynyszyn, L.A., Maher, E.J., & Corwin, T.W. (2011). Getting with the (evidence-based) program: An evaluation of the Incredible Years Parenting Training Program in child welfare. *Children & Youth Services Review,* 33, 747-757.

Mitchell, G., Absler, D., & Humphreys, C. (2015). "She's just like me": The Role of the Mentor with Vulnerable Mothers and their Infants. *Children Australia,* 40, 33-42.

Nabuco, M.E., Aguiar, M.S., Costa, C., & Morais, D. (2014). Evaluation of the effectiveness of the implementation of the A PAR parental intervention programme in Portugal. Child development and parenting support. *European Early Childhood Education Research Journal,* 22, 554-572.

Pancer, S.M., Nelson, G., Hasford, J., & Loomis, C. (2013). The Better Beginnings, Better Futures Project: Long-term Parent, Family, and Community Outcomes of a Universal, Comprehensive, Community-Based Prevention Approach for Primary School Children and their Families. *Journal of Community & Applied Social Psychology,* 23, 187-205.

Parcel, T.L., & Pennell, J. (2012). Child and Family Teams Building Social Capital for At-Risk Students: A Research Note. pp. 75-91).

Romijnders, K.A., Wilkerson, J.M., Crutzen, R., Kok, G., Bauldry, J., & Lawler, S.M. (2017). Strengthening Social Ties to Increase Confidence and Self-Esteem Among Sexual and Gender Minority Youth. *Health Promot Pract,* 18, 341-347.

Schwartz, S., Rhodes, J., Spencer, R., & Grossman, J. (2013). Youth Initiated Mentoring: Investigating a New Approach to Working with Vulnerable Adolescents. *American Journal of Community Psychology,* 52, 155-169.

Stubbs, J.M., & Achat, H.M. (2016). Sustained health home visiting can improve families' social support and community connectedness. *Contemp Nurse,* 52, 286-299.

Swenson, C.C., Schaeffer, C.M., Henggeler, S.W., Faldowski, R., & Mayhew, A.M. (2010). Multisystemic Therapy for Child Abuse and Neglect: a randomized effectiveness trial. *J Fam Psychol,* 24, 497-507.

Valdez, C.R., Mills, C.L., Barrueco, S., Leis, J., & Riley, A.W. (2011). A Pilot Study of a Family-Focused Intervention for Children and Families Affected by Maternal Depression. *J Fam Ther,* 33, 3-19.

Valdez, C.R., Padilla, B., Moore, S.M., & Magana, S. (2013). Feasibility, acceptability, and preliminary outcomes of the Fortalezas Familiares intervention for latino families facing maternal depression. *Fam Process,* 52, 394-410.

van Dam, L., Neels, S., de Winter, M., Branje, S., Wijsbroek, S., Hutschemaekers, G., et al. (2017). Youth Initiated Mentors: Do They Offer an Alternative for Out-of-Home Placement in Youth Care? *British Journal of Social Work,* 47, 1764-1780.

Van Voorhees, B.W., Vanderplough-Booth, K., Fogel, J., Gladstone, T., Bell, C., Stuart, S., et al. (2008). Integrative internet-based depression prevention for adolescents: a randomized clinical trial in primary care for vulnerability and protective factors. *J Can Acad Child Adolesc Psychiatry,* 17, 184-196.

Vazquez, N., Molina, M.C., Ramos, P., & Artazcoz, L. (2017). Effectiveness of a parent-training program in Spain: reducing the Southern European evaluation gap. *Gac Sanit*.

Vella, S.A., Swann, C., Batterham, M., Boydell, K.M., Eckermann, S., Fogarty, A., et al. (2018). Ahead of the game protocol: a multi-component, community sport-based program targeting prevention, promotion and early intervention for mental health among adolescent males. *BMC Public Health,* 18, 390.
