# Supplementary material for: Mobilising social support to improve mental health for children and adolescents: A systematic review using principles of realist synthesis
Source: PLoS One. 2021 May 20;16(5):e0251750. doi: 10.1371/journal.pone.0251750 (PMC8136658; doi:10.1371/journal.pone.0251750)
Supplement: Attachment — Submitted filename: Response to reviewers final.docx [file pone.0251750.s009.docx]

| **Editor’s comments** | **Our response** |
| --- | --- |
| 1. Please ensure that your manuscript meets PLOS ONE's style requirements, including those for file naming.  The PLOS ONE style templates can be found at  <https://journals.plos.org/plosone/s/file?id=wjVg/>  PLOSOne_formatting_sample_main_body.pdf    and  <https://journals.plos.org/plosone/s/file?id=ba62/>  PLOSOne_formatting_sample_title_authors_affiliations.pdf | We changed the formatting style so that the manuscript meets the requirements. |
| 2. Please review your reference list to ensure that it is complete and correct. If you have cited papers that have been retracted, please include the rationale for doing so in the manuscript text, or remove these references and replace them with relevant current references. Any changes to the reference list should be mentioned in the rebuttal letter that accompanies your revised manuscript. If you need to cite a retracted article, indicate the article’s retracted status in the References list and also include a citation and full reference for the retraction notice. | We checked the reference list accordingly. |
| 3. In your Data Availability statement, you have not specified where the minimal data set underlying the results described in your manuscript can be found. PLOS defines a study's minimal data set as the underlying data used to reach the conclusions drawn in the manuscript and any additional data required to replicate the reported study findings in their entirety. All PLOS journals require that the minimal data set be made fully available. For more information about our data policy, please see <http://journals.plos.org/plosone/s/data-availability>.  Upon re-submitting your revised manuscript, please upload your study’s minimal underlying data set as either Supporting Information files or to a stable, public repository and include the relevant URLs, DOIs, or accession numbers within your revised cover letter. For a list of acceptable repositories, please see http://journals.plos.org/plosone/s/data-availability#loc-recommended-repositories. Any potentially identifying patient information must be fully anonymized.  Important: If there are ethical or legal restrictions to sharing your data publicly, please explain these restrictions in detail. Please see our guidelines for more information on what we consider unacceptable restrictions to publicly sharing data: http://journals.plos.org/plosone/s/data-availability#loc-unacceptable-data-access-restrictions. Note that it is not acceptable for the authors to be the sole named individuals responsible for ensuring data access.    We will update your Data Availability statement to reflect the information you provide in your cover letter. | We submitted the following data availability statement:  “The authors confirm that the data supporting the findings of this study are available within the article [and/or] its supplementary materials.”  We adhere to the best practices for reporting by following the Reporting Guidelines for Systematic reviews as specified by the journal <https://journals.plos.org/plosone/s/best-practices-in-research-reporting>;   - We included the PRISMA checklist. - We refer in the method section to the protocol registered at Prospero. |
| 4. Please amend either the abstract on the online submission form (via Edit Submission) or the abstract in the manuscript so that they are identical. | We updated the abstracts online and in the manuscript and ensured they are identical. |
| 5. Please include captions for your Supporting Information files at the end of your manuscript, and update any in-text citations to match accordingly. Please see our Supporting Information guidelines for more information: http://journals.plos.org/plosone/s/supporting-information | We included captions for the Supporting Information files according to the guidelines and updated in-text citations to match those. |

| **Reviewers’ comments** | **Our response** |
| --- | --- |
| Reviewer #1: This is an interesting review and clearly a lot of work has clearly gone into this SR review. The tables (especially those in the supplement material) are very helpful and add to the current literature.  A few minor suggestions below. | We thank the reviewer for this positive feedback, and the suggested edits which we think has improved the quality of the paper. |
| Methods:  Line 177: Study rigor was assessed in relation to study design, samples size, data collection and outcomes, etc. but no detail on what gets you a LOW v HIGH. Please provide a bit more detail on the rating system | We thank the reviewer for this suggestion. We added some detail about the tool and the rating system.  “Following the guidance and algorithm provided by the tool, we applied ‘low’, ‘high’ and, where information was insufficient to rate the criterion, ‘can’t tell’ ratings. The algorithm provides quality criteria as well as examples of how to apply those for qualitative, quantitative randomised controlled trails, quantitative non-randomised controlled trials, quantitative descriptive studies and mixed-method studies. Studies were not excluded based on relevance or rigour.” |
| Lines 185: I don’t quite understand what this means? Do you mean if the inclusion was 5-12 y/o the study would land in the 3 to 9 category? I think if you remove the “mean” ages from this and just include age range in years given you don’t really calculate a mean. | We thank the reviewer for this comment and suggestion. We agree that the term ‘mean’ and the wording to describe how we applied categories was misleading. We changed the text in line with the reviewer’s suggestion to make this clear.  “Age categories included infants aged 0 to 2 years, children aged 3 to 9 years, and adolescents aged 10 to 18 years. For studies where the age range fell between two categories, they landed in the category that captured more years e.g. if the inclusion was 5 to 12 years, the study would land in the 3 to 9 years category.” |
| Grammar: move the common from 2, to after years, and add years after each category)  Age categories included mean ages of infants 0 to 2, years children aged 3 to 9 and adolescents aged 10 to 18. For studies, where the age range fell between two categories, they were moved to the category in which the majority of children would fall under. | We thank the reviewer for spotting the error. Also addressing the comment above, the passage now reads as follows.  “Age categories included infants aged 0 to 2 years, children aged 3 to 9 years, and adolescents aged 10 to 18 years. For studies where the age range fell between two categories, they landed in the category that captured more years e.g. if the inclusion was 5 to 12 years, the study would land in the 3 to 9 years category.” |
| Lines 189: I don’t quite understand this sentence. What does context-mechanisms outcomes configurations for components of interventions mean?  Could you also provide a hypothesized mechanism of change figure or something to make this a bit more clear regarding the relationship you were hoping to evaluate and mechanisms or pathways you were hoping to be explored in the interventions you identified?  By identifying data patterns, a realist synthesis seeks to derive context-mechanisms-outcomes configurations for components of interventions, which explain how the relationship between resource inputs, human reaction processes, and contextual factors lead to  particular outcomes. | We thank the reviewer for this comment and helpful suggestion.  One, we decided to remove the term context-mechanisms-outcomes configurations as we realised it would require additional explanation in order to be helpful, and the added value was limited, especially as we are not developing those in the paper, and it is not required for subsequent understanding of text.  Two, we changed the wording to make it clearer which pathways we were hoping to explore. We changed the text to:  “By identifying data patterns, a realist synthesis seeks to derive information about relationships between resource inputs, human reaction processes, and contextual factors for interventions or intervention components, and how those lead to particular outcomes. In this paper, our main interest was to understand how social support was conceptualised, e.g. with regard to types of social support, which changes in human interaction processes were assumed to be required in order to improve children’s mental health outcomes, and how those were modelled into the intervention design. We used the above-mentioned dimensions (intervention components, context, mechanisms, and outcomes) for the synthesis of the data and we present findings by research questions.” |
| Line 197 should be ‘of’ not ‘if’ provide details of the studies including the details of how assessments if study relevance and quality were derived. | We thank the reviewer for spotting this error. We corrected this. |
| Line 281 remove the e.g. before the reference? | We thank the reviewer for this suggestion. We replaced “e.g.” with “see for example…” |
| review also suggest that in order for interventions to be effective, they might need to BE population and context specific,  ADD the word BE before population | We thank the reviewer for spotting this error. We corrected this. |
